# Evolutionary analysis of hydrophobin gene family in two wood-degrading basidiomycetes, *Phlebia brevispora* and *Heterobasidion annosum* s.l.

**DOI:** 10.1186/1471-2148-13-240

**Published:** 2013-11-04

**Authors:** Anthony C Mgbeahuruike, Andriy Kovalchuk, Hongxin Chen, Wimal Ubhayasekera, Fred O Asiegbu

**Affiliations:** 1 Department of Forest Sciences, University of Helsinki, Helsinki, Finland; 2 Department of Molecular Biology, Swedish University of Agricultural Sciences (SLU), Uppsala, Sweden

**Keywords:** Hydrophobins, Gene evolution, Basidiomycetes, Ascomycetes, Pathogen

## Abstract

**Background:**

Hydrophobins are small secreted cysteine-rich proteins that play diverse roles during different phases of fungal life cycle. In basidiomycetes, hydrophobin-encoding genes often form large multigene families with up to 40 members. The evolutionary forces driving hydrophobin gene expansion and diversification in basidiomycetes are poorly understood. The functional roles of individual genes within such gene families also remain unclear. The relationship between the hydrophobin gene number, the genome size and the lifestyle of respective fungal species has not yet been thoroughly investigated. Here, we present results of our survey of hydrophobin gene families in two species of wood-degrading basidiomycetes, *Phlebia brevispora* and *Heterobasidion annosum* s.l. We have also investigated the regulatory pattern of hydrophobin-encoding genes from *H. annosum* s.s. during saprotrophic growth on pine wood as well as on culture filtrate from *Phlebiopsis gigantea* using micro-arrays. These data are supplemented by results of the protein structure modeling for a representative set of hydrophobins.

**Results:**

We have identified hydrophobin genes from the genomes of two wood-degrading species of basidiomycetes, *Heterobasidion irregulare*, representing one of the microspecies within the aggregate *H. annosum* s.l., and *Phlebia brevispora*. Although a high number of hydrophobin-encoding genes were observed in *H. irregulare* (16 copies), a remarkable expansion of these genes was recorded in *P. brevispora* (26 copies). A significant expansion of hydrophobin-encoding genes in other analyzed basidiomycetes was also documented (1–40 copies), whereas contraction through gene loss was observed among the analyzed ascomycetes (1–11 copies). Our phylogenetic analysis confirmed the important role of gene duplication events in the evolution of hydrophobins in basidiomycetes. Increased number of hydrophobin-encoding genes appears to have been linked to the species’ ecological strategy, with the non-pathogenic fungi having increased numbers of hydrophobins compared with their pathogenic counterparts. However, there was no significant relationship between the number of hydrophobin-encoding genes and genome size. Furthermore, our results revealed significant differences in the expression levels of the 16 *H. annosum s.s.* hydrophobin-encoding genes which suggest possible differences in their regulatory patterns.

**Conclusions:**

A considerable expansion of the hydrophobin-encoding genes in basidiomycetes has been observed. The distribution and number of hydrophobin-encoding genes in the analyzed species may be connected to their ecological preferences. Results of our analysis also have shown that *H. annosum* s.l. hydrophobin-encoding genes may be under positive selection. Our gene expression analysis revealed differential expression of *H. annosum* s.s. hydrophobin genes under different growth conditions, indicating their possible functional diversification.

## Background

Hydrophobins are surface-active proteins produced by filamentous fungi [1,2]. They are small secreted proteins with eight cysteine residues arranged in a strictly conserved motif [3,4]. The cysteine residues form four disulfide bridges connecting beta strands and stabilizing the protein structure. Another important feature of hydrophobins is their ability to self-assemble into amphiphilic films at hydrophilic/hydrophobic interfaces [5-7]. Hydrophobins have been reported in filamentous fungi belonging to the phyla Ascomycota and Basidiomycota [8]. They are often secreted extracellularly but can also be found inside fungal structures such as fruiting bodies and hyphae [9]. Based on solubility and sequence characteristics, hydrophobins can be classified into two major classes: Class I and Class II [1,10]. Although the two classes are similar in many ways, class I hydrophobins have larger size and more diversity in amino acid sequence than class II [1,10]. Hydrophobins are expressed at different stages of fungal life cycle: sporulation, fruiting body formation and during growth of vegetative hyphae [9]. Studies have shown that hydrophobins play important role in fungal pathogenesis where they act as virulence factors to enhance fungal infection [11-15]. They have also been reported to be involved in the attachment of fungal structures [16,17] and the emergence of aerial hyphae from submerged conditions [18-20]. Hydrophobins have been implicated in diverse fungal interactions such as symbiosis [21], mycorrhiza formation [22], and antagonistic interactions [20,23]. Evidence of hydrophobin involvement in cell wall assembly during pathogenic interactions where the monomers act as elicitors and toxins have been reported [24]. Recent gene expression studies have shown that *P. gigantea* hydrophobin encoding genes 1 and 2 (*Pgh1* and *Pgh2*) are highly transcribed in the interaction zone between the biological control agent *P. gigantea* and the tree pathogen *H. annosum* s.l. [20,23]. However, the actual roles of these genes in the interaction are not known. Previous studies have demonstrated a high level of sequence divergence in *P. gigantea* hydrophobin-encoding genes *Pgh1* and *Pgh2*[20] and the hydrophobins from the pathogenic fungus *H. irregulare Hah1* and *Hah2*[12]. These observations raised crucial questions on the evolutionary forces driving the rapid differentiation of this gene family. Available data also indicate a considerable amount of variation in the numbers of hydrophobin-encoding genes across other fungal taxa, ranging from 1 gene in *Acremonium alcalophilum* to 40 genes in *Trametes versicolor*. This may suggest that hydrophobin genes could be under a dynamic evolutionary process across most fungal taxa.

Gene duplication is an important evolutionary process that plays a crucial role in an organism’s complexity, adaptation and diversification to closely related strains and species [25,26]. Reports have shown that duplication of genes results in functional diversification and gene expression patterns observed in different fungi and other organisms [27]. Paralogous genes resulting from duplication events create genetic redundancy, which may be vulnerable to selection pressure [25,27]. Genome-wide analysis of gene duplication has shown that this biological phenomenon occurs at a very high rate. However, the fate of duplicated genes and the forces driving their fixation and divergence still remain unknown [26]. There has been increasing number of evidences showing that mutated genes with deleterious effects are purged from the genome through purifying selection whereas copies with enhanced functions are fixed in the population through positive or diversifying selection [28,29]. Furthermore, evidence of duplication and losses among stress-related genes has been documented in the literature whereas growth-related genes have been shown to be selected against change in the copy number [30]. Gene contraction through gene loss and expansion through duplication are common processes in gene family evolution and it has been documented in chitinases [31] and glycosyl hydrolases (GH28) [32] gene families. However, the mechanisms driving these evolutionary processes in fungal hydrophobins are poorly understood.

In this study, we investigated hydrophobin gene family evolution in several diverse fungal groups, we also analyzed recombination events in *H. annosum* s.l. by examining the ratio of non-synonymous (dN) to synonymous substitutions per site (dS). We tested correlation between number of hydrophobin-encoding genes, overall genome size and their ecological strategy. The transcript abundance of sixteen hydrophobin genes from *H. annosum* s.s. during saprotrophic growth on pine wood as well as on culture filtrate from *P. gigantea* was further evaluated using micro-array. The results from this study have further highlighted the possible involvement of hydrophobin genes in fungal ecological lifestyle.

## Results

### Hydrophobin sequence identification and alignment

#### *Sequence alignment of selected fungal hydrophobins*

**Figure 1:**
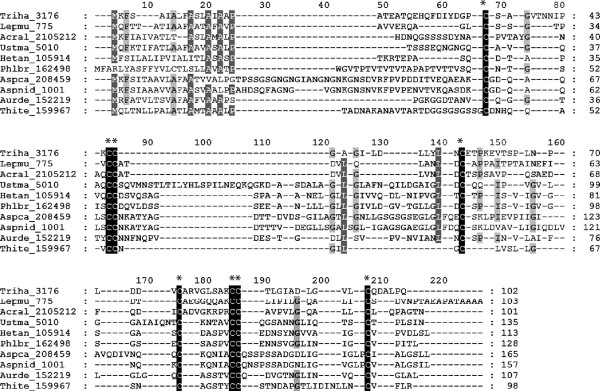
**Sequence alignment of selected fungal hydrophobins.** Alignment of amino acid sequences of several selected fungal hydrophobins representing both known classes and including two deviating sequences from *U. maydis* and *T. terrestris*. Following abbreviations are used to indicate the species of origin (in alphabetic order): Acral, *Acremonium alcalophilum*; Aspca, *Aspergillus carbonarius*; Aspnid, *Aspergillus nidulans*; Aurde, *Auricularia delicata*; Hetan, *H. irregulare*; Lepmu, *Leptosphaeria maculans*; Phlbr, *Phlebia brevispora*; Thite, *Thielavia terrestris*; Triha, *Trichoderma atroviride*; Ustma, *Ustilago maydis*. Sequences from *A. carbonarius* (jgi|Aspca3|208459|fgenesh_isot), *A. nidulans* (jgi|Aspnid1|1001|AN8803), *A. delicata* (jgi|Aurde1|152219|estExt_fgene), *P. brevispora* (jgi|Phlbr1|162498|estExt_Genem) and *H. irregular*e (jgi|Hetan2|105914|Hetan1.Genem) belong to the Class I; sequences from *L. maculans (*jgi|Lepmu1|775|Lema_T007750.1), *T. atroviride* (jgi|Triha1|3176|gm1.3176_g) and *A. alcalophilum* (jgi|Acral2|2105212|e_gw1.4.137) represent the Class II. *T. terrestris* (jgi|Thite2|159967|Thite1.genem) and *U. maydis* (jgi|Ustma1|5010|UM05010) deviated from the general consensus of classes I and II. Conserved cysteine residues are shaded black and indicated with an asterisk; functionally similar residues are shaded grey. Alignment was produced with MUSCLE alignment tool in MEGA 5.0.

Alignment of the manually curated sequences of hydrophobin encoding genes from representative fungal species screened in this study showed eight conserved cysteine residues necessary for disulfide bridge formation, a characteristic feature of all fungal hydrophobins (Figure 1). There was high sequence diversity in the multiple alignments. A comparison was made between the aligned sequences and already published sequence consensus of class I, C-X_5-7_-C-C-X_19-39_-C-X-_8–23_-C-X_5_-C-C-X_6-18_-C-X_2-13_[33] and class II, C-X_9_-C-C-X_11_-C-X_14_-_16_-C-X_8_-C-C-X_10_-C-X_6-7_[14] hydrophobins. Based on the consensus, sequences were clearly separated into two different groups, class I and class II. Hydrophobins from *H. irregulare* and *P. brevispora* were found to be class I members. There was a long stretch of amino acids (aas), 26–39 amino acid residues between the C3/C4 position in the hydrophobins from the class I proteins*.* However, class II hydrophobins showed a short stretch of amino acids at this region (C3/C4) with all members investigated in this study having 11 aa at position C3/C4 (Table 1). The hydrophobins from the thermophilic fungus *Thielavia terrestris* and the corn smut fungus *Ustilago maydis* deviated from the remaining analyzed hydrophobins in the length of the region between cysteine residues C3 and C4 (Figure 1; Table 1). The hydrophobin from *T. terrestris* had very short C3/C4 stretch consisting of only 5 amino acids residues, while the one from *U. maydis* had unusually long C3/C4 regions of 49 amino acid residues.

**Table 1:** Spacing between conserved cysteine residues in different classes of hydrophobins

**Class**	**Numbers of amino acid residues between conserved cysteine residues**				
	**C1/C2**^**a**^	**C3/C4**	**C4/C5**	**C5/C6**	**C7/C8**
Class I (basidiomycetes)	6	26–33	12–13	6	13
Class I (ascomycetes)	6–7	26–39	18–21	6–8	15–17
Class II	9–10	11	15–16	2–7	10
*T. terrestris* protein 159967	7	5	8	5	12
*U. maydis* protein 5010	6	49	17	5	16

^a^ = Positions of conserved cysteine residues. Cysteine residues C2/C3 and C6/C7 are adjacent in all characterized hydrophobins.

### Domain structure, hydropathy pattern and homology modeling of selected hydrophobins

**Figure 2:**
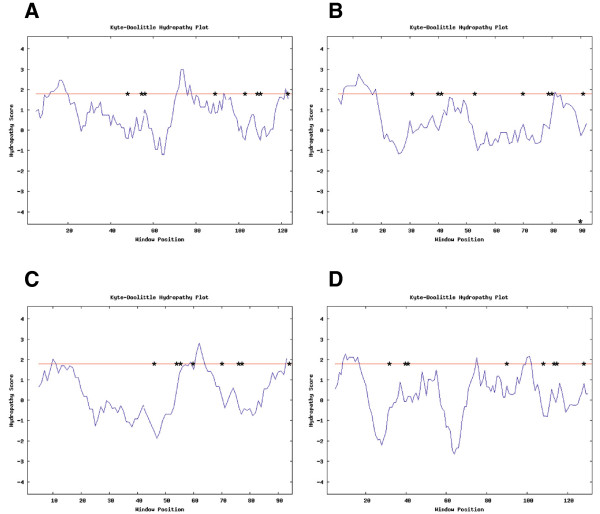
**Hydropathy plot of different classes of fungal hydrophobins. (A)** Class I hydrophobin from *Phlebia brevispora, ***(B)** Class II hydrophobin from *N. tetrasperma, ***(C)** hydrophobin from *T. terrestris* and **(D)** hydrophobin from *U. maydis.* Hydropathy plot was made with Kyte-Doolittle hydropathy plot, Version 2.0u66 in Windows 9.0. Hydrophobic amino acids show more positive peaks whereas hydrophilic amino acids show more negative peaks. Strong negative peaks show possible surface regions of globular proteins, while peaks above the threshold line indicate possible transmembrane regions of the proteins. Positions of conserved cysteine residues are indicated with astersisks.

**Figure 3:**
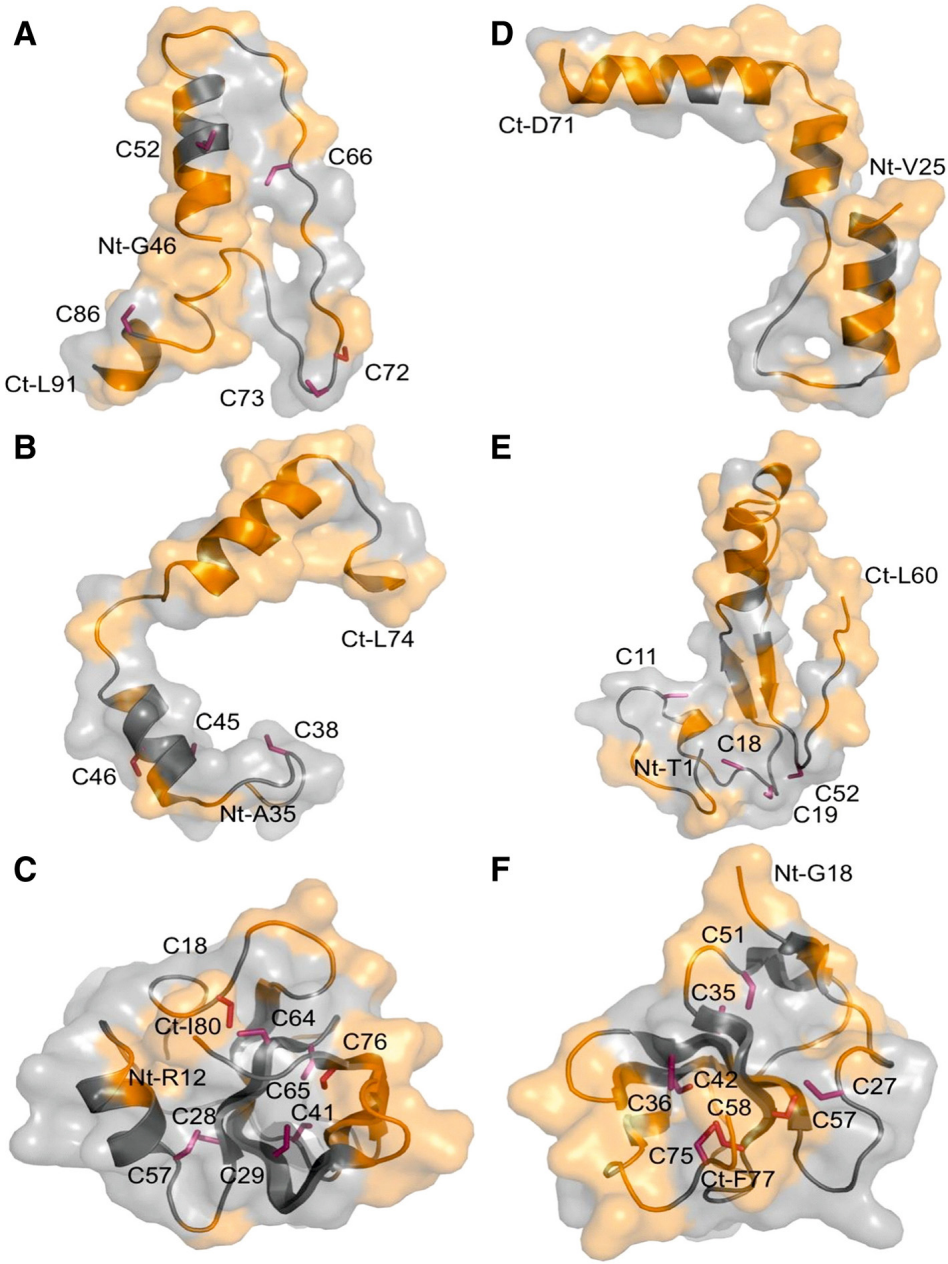
**Homology modeling of hydrophobins.** Surface and ribbon models of the parts of the hydrophobins from **(A) ***P. gigantea* (protein ID 104621), **(B) ***H. irregulare* (protein ID 181098), **(C) ***V. dahliae* (protein ID 6939), **(D) ***U. maydis* (protein ID 5010), **(E) ***L. bicolor (*protein ID 473162), and **(F) ***T. terrestris* (gm1.6178_g, protein ID 2089872). The protein IDs correspond to the protein model numbers in the Fungal Genomic Platform database at JGI. The hydrophobic areas and conserved cysteine residues are shown in orange and hotpink respectively. N- and C-termini (Nt- and Ct-) are shown with the respective amino acids.

A comparison of the domain structures of hydrophobins from a subset of the fungal species screened in this study was made to determine if the differences in their ecological habits could be explained by different domain patterns. Furthermore, the hydropathy profile of hydrophobins from class I was compared with the profile from class II members as well as hydrophobins from *T. terrestris* and *U. maydis* (Figure 2A-D). Class I hydrophobins showed higher hydrophobicity stretch (positive values) (Figure 2A), when compared with the pattern from class II members (Figure 2B). The cysteine doublets in the Class I hydrophobins are followed by a long stretch of hydrophilic residues, whereas in the proteins belonging to the Class II, the cysteine doublets were followed by hydrophobic residues. For *U. maydis* and *T. terrestris*, the hydropathy patterns were similar to that of class I proteins (Figure 2C-D). The signal peptides of the hydrophobins screened in this study lie in the region of the first 21 amino acids, although in some cases the signal peptide was missing. Homology modeling of a subset of hydrophobins sequences revealed the distribution of the hydrophobic residues and conserved cysteine residues (Figure 3). This clearly shows that the residues are arranged as patches. The models only cover parts of the relevant hydrophobins, as construction of complete models was hindered by the lack of template structures.

### Phylogenetic analysis of the fungal hydrophobins and ecological strategy

#### Basidiomycetes and ascomycetes

In order to understand the evolutionary relationships of the fungal hydrophobins from both ascomycetes and basidiomycetes screened in this study (Additional file 1: Table S1), species based trees were reconstructed using the NJ method with JTT matrix-based model [34]. Two distinct separations along the two classes of hydrophobins were evident, all the class I hydrophobins clustered in clade A whereas class II members grouped together in clade B (Additional file 2: Figure S1). Other subclades such as C, D, E, F and G were evident. Subclade C is a mixed group containing class I proteins from both ascomycetes and basidiomycetes. Most of the basidiomycetes in this group are from the order Polyporales except few species like *Auricularia delicata* and *Schizophyllum commune* which were from the orders Auriculariales and Agaricales, respectively. It was also interesting to see that all the hydrophobins from *A. delicata,* a basidiomycete, are closer related to the class I proteins from the ascomycetes than to the remaining members of class I hydrophobins from basidiomycetes (Additional file 2: Figure S1, subclade C). Hydrophobins from *U. maydis* and *T. terrestris* which showed a deviation from the general consensus of classes I and II (49 aas at C3/C4 and 5 aa at C3/C4) respectively, were nested within the Class I hydrophobins (Additional file 2: Figure S1) in our phylogenetic analysis and thus, most likely, they are just two extreme examples of the variation in length of C3/C4 region in the Class I hydrophobins. Furthermore, the hydrophobin from *T. terrestris* with unusually short stretch of amino acids between cysteine residues C3 and C4 appeared within group C. Subclade D contains white rot fungi of the order Polyporales except *Wolfiporia cocos*, a brown rot fungus. Most of the hydrophobins from *P. brevispora* clustered in this group. However sequences of hydrophobins from *H. irregulare* could not be resolved into a distinct clade. Groups E, F and G consisted of class I hydrophobins from basidiomycetes of different systematic positions and ecological lifestyles (brown rots, white rots, mycorrhiza-formers and saprotrophs). Another interesting observation from the phylogenetic grouping is that many ascomycetes such as *Magnaporthe grisea*, *Trichoderma virens*, *Neurospora discreta*, *Neurospora crassa* and *Neurospora tetrasperma* appeared to have both Classes I and II proteins and the two classes separated into different clades (Additional file 2: Figure S1)*.*

#### Basidiomycetes

A similar trend was observed for the phylogenetic tree involving only hydrophobins from basidiomycetes. Seven main clades and two smaller clades were formed (Additional file 3: Figure S2). Members of clades H, I and J were mostly hydrophobins from fungi of the order Polyporales, all the fungi in this group except *W. cocos* and *Punctularia strigosozonata* are white rotters. Most of the sequences of hydrophobins from *P. brevispora* were resolved in clade J. However, sequences of hydrophobins from *H. irregulare* have not formed a distinct clade. Clade K consists of hydrophobins from fungi of diverse systematic positions (orders Agaricales, Polyporales, Russulales, Corticiales and Boletales) and ecological strategies. Clades L and P are relatively smaller clades with hydrophobins from fungi representing different orders and lifestyles. In clade M, sequences of hydrophobins from *S. commune*, a white rot fungus dominated the group with hydrophobins from *A. delicata.* Group N is a relatively small group comprising of hydrophobins from fungal species of the orders Agaricales, Boletales and Dacrymycetales. Fungal species in this group have mixed lifestyle, while some are saprotrophic in nature (*Coprinopsis cinerea*), others are brown rotters. In addition, some sequences of hydrophobins from the mycorrhizal fungus, *Laccaria bicolor* were found in this clade. In clade O, all the hydrophobin sequences separated into fungal species with brown rotting habits. The fungal species (*Coniophora puteana* and *Serpula lacrymans*) in this group are from the order Boletales.

#### Ascomycetes

**Figure 4:**
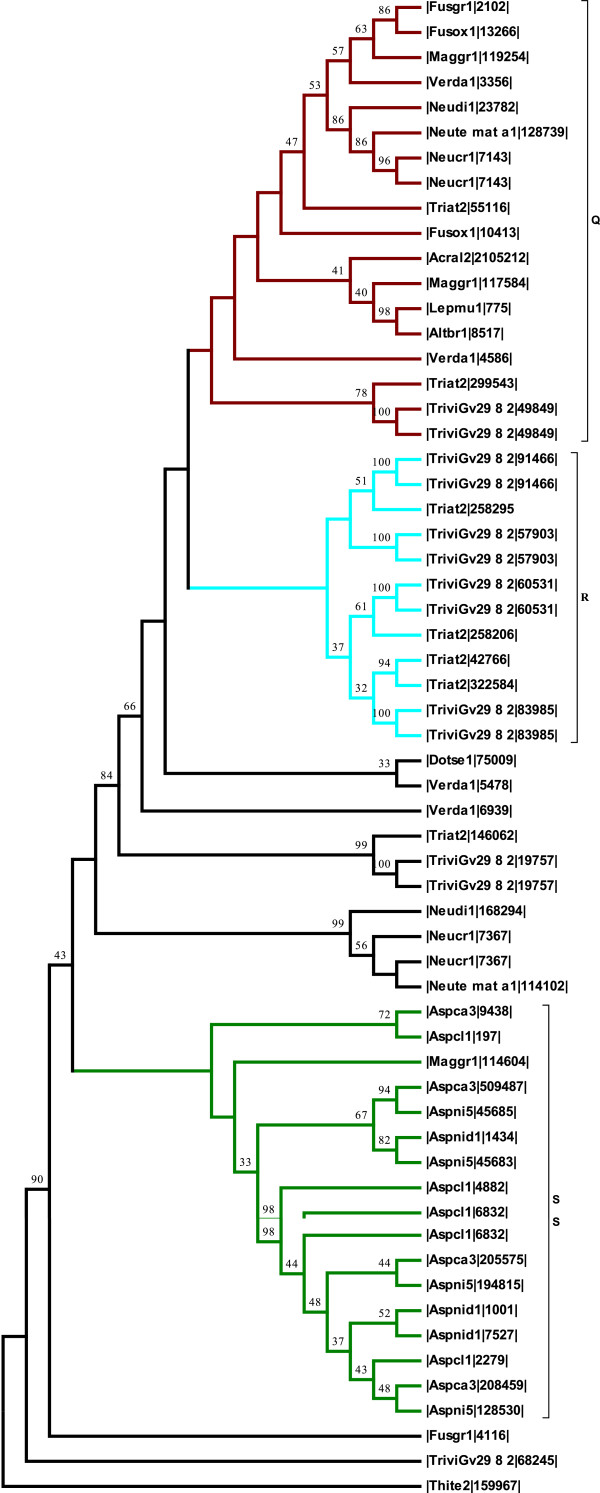
**Phylogenetic tree showing the relationship between hydrophobins from a representative set of ascomycetes.** The tree was inferred using the Neighbor-Joining method. The optimal tree with the sum of branch length = 23.48723281 is shown. The percentage of replicate trees in which the associated taxa clustered together in the bootstrap test (1000 replicates) are shown above the branches. The tree is drawn to scale, with branch lengths in the same units as those of the evolutionary distances used to infer the phylogenetic tree. The evolutionary distances were computed using the JTT matrix-based method and are in the units of the number of amino acid substitutions per site. The analysis involved 59 amino acid sequences. All ambiguous positions were removed for each sequence pair. There were a total of 136 positions in the final dataset. Three clades **Q** (purple), **R** (blue) and **S** (green) are represented in the tree. Clades **R** and **S** = monophyletic groups. Clade **Q** = class I hydrophobins from ascomycetes with mixed lifestyles, pathogens, saprotrophs and mycoparasites*.* Clades **R** = class II hydrophobins from *Trichoderma* species. Clade **S** = class II hydrophobins from *Aspergillus* species except one sequence from the rice blast fungus, *M. grisea*. Fungal species are indicated with the following abbreviations. *|Thite2|, Thielavia terrestris; |Acral2|, Acremonium alcalophilum*, |Aspca3|, *Aspergillus carbonarius, |Aspcl1|, Aspergillus clavatus*; |Aspnid1|, *Aspergillus nidulans*; |Dotse1|, *Dothistroma septosporum, |*Lepmu1*|, Leptosphaeria maculans*, |Triat2| *Trichoderma atroviride*, |TriviGv|, *Trichiderma virens*, |Altbr1|, *Alternaria brassicicola*, |Triha1|, *Trichoderma harzianum*, |Pench1|, *Penicillium chrysogenum*, |Neudi1|, *Neurospora discreta*, |Fusgr1|, *Fusarium graminearum*, |Fusox1|, *Fusarium oxysporum*, |Maggr1|, *Magnaporthe grisea*, |Neucr1|, *Neurospora crassa*, |Neute_mat_a1|, *Neurospora tetrasperma,* |Verda1|, *Verticillium dahliae*.

Three major clades Q, R and S were evident in the phylogenetic tree reconstructed with the ascomycetes (Figure 4). The branches were strongly supported with relatively high boothstrap values. Clades R and S formed monophyletic groups with members of the group being class II hydrophobins from *Trichoderma* and *Aspergillus* species respectively, except one sequence of hydrophobin from *M. grisea* that nested with the members of clade S. Clade Q is a mixed clade with class I proteins from different fungal species. Members of the group include sequences of hydrophobins from the pathogenic fungi (*Fusarium oxysporum*, *M. grisea, Verticillium dahliae*, *Leptosphaeria maculans* and *Alternaria brassicicola*). Other members of the group are hydrophobins from the saprotrophic fungi (*Neurospora* spp. and *A. alcalophilum)* and the mycoparasitic fungi*, Trichoderma* species*.*

### Distribution, genome size and hydrophobin gene family evolution in ascomycetes and basidiomycetes

**Figure 5:**
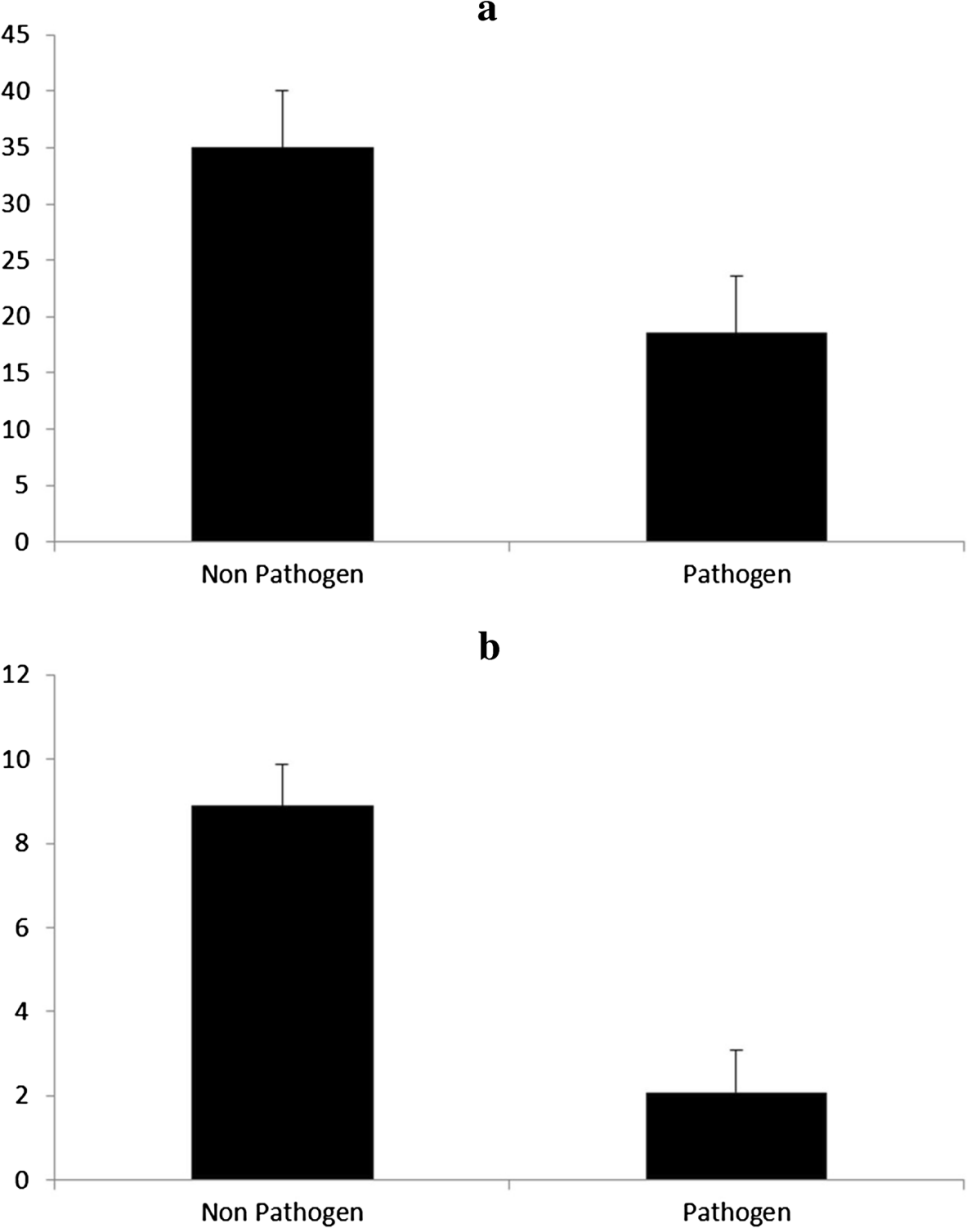
**Relationships between genome sizes, hydrophobin gene copy numbers and ecological strategies of selected fungal species. a)** Relationships between genome size and different ecological strategies. The bars indicate average genome size in the analyzed pathogenic and non-pathogenic fungi. **b)** Relationships between hydrophobin copy number and different ecological strategies. The bars indicate average genome size in the analyzed pathogenic and non-pathogenic fungi.

A survey of the distribution of hydrophobin-encoding genes and genome sizes of the fungi analyzed in this study revealed considerable variation in the copy number of hydrophobin genes ranging from 1 in *A. alcalophilum* to 40 copies in *T. versicolor* (Additional file 4: Figure S3 and Additional file 5: Figure S4, Additional file 1: Table S1). There were 26 functional copies of hydrophobin-encoding genes in *P. brevispora* with a genome size of 49.96 MB. Although previous studies have reported 13 copies of hydrophobin encoding genes in *H. irregulare*[35], our analysis revealed 24 predicted gene copies of hydrophobins in the genome of *H. irregularre,* out of this number, only 16 are functional proteins*.* This difference in the number of hydrophobin-encoding genes observed in *H. irregulare* could be a result of automatic annotation problem. However, among the basidiomycetes screened in this study, hydrophobins were completely absent in the *Pucciniales.* The absence of hydrophobins in rust fungi may be either linked with their life style or with their relatively simple life forms (i.e., relatively simple morphology, absence of massive fruiting bodies). The second explanation seems more probable as hydrophobins are missing both in parasitic (*Puccinia*) and free-living (*Rhodotorula*) members of Pucciniomycotina. In the ascomycetes group, there was no evidence of hydrophobins in all the *Saccharomycetales*/yeast screened in our study (Additional file 1: Table S1). Also the genome size was variable across the different fungal species screened, ranging from 11.5 MB in *Pichia membranifaciens* (yeast) to 101.1 MB in *Melampsora laricis-populina*, a basidiomycete with biotrophic lifestyle (Additional file 1: Table S1). A considerable expansion of hydrophobin-encoding genes was observed in basidiomycetes (*P =* 0.002) while a contraction of the same gene family was evident in the ascomycetes. A comparison of the genome size and the number of hydrophobin encoding genes of a subset of the fungal isolates used in this study was made using Pearson’s partial correlation, although a correlation between genome size and the number of hydrophobin-encoding genes exist, it was not statistically significant (*R*^*2*^ = 0. 135, *P* = 0.35). Furthermore, there was no significant relationship between genome size and ecological strategy (Figure 5a), however, there was a statistically significant relationship between ecological strategy (non-pathogenic) and the number of hydrophobin-encoding genes (Figure 5b). The fungi with non-pathogenic lifestyle tend to be more favored by higher numbers of hydrophobin-encoding genes (*P =* 0.0001). However, fungi with pathogenic lifestyles showed no statistically significant relationship between the number of hydrophobin-encoding genes and their ecological strategies.

### Gene clusters and inventory of hydrophobins in *P. brevispora* and *H. irregulare* hydrophobins

The 26 class I hydrophobin-encoding-genes found in the genome of *P. brevispora*, were arranged in a relatively clustered pattern (Additional file 6: Table S2). A good number of the proteins (6) were located in scaffold 12, whereas 4 proteins each where found in scaffolds 11 and 14 respectively. Scaffold 23 had 3 proteins, while scaffolds 9, 14 and 19 contained 2 proteins each. Other scaffolds, 75, 30 and 38 had one protein each.

In *H. irregulare*, 24 putative class I hydrophobin encoding gene sequences were identified, 16 appeared to be functional proteins whereas 8 were probable pseudogenes. A similar clustering pattern of hydrophobin-encoding genes was observed in the genome of *H. irregulare* (Additional file 7: Table S3), most of the genes (15) clustered in scaffold 11. Scaffold 6 had 3 genes, whereas 2 genes were found in scaffold 3. Scaffolds 4, 8 and 9 had 1 gene each.

### Selection tests for *H. irregulare* hydrophobins

A selection test was carried out in a subset of hydrophobins from *H. irregulare*. A high dN/dS ratio (>1) was observed in all the tested hydrophobin genes*.* This result was supported by the *D* test statistics (Table 2). Only one recombination event was observed in *H. irregular*e.

**Table 2:** **Selection test for hydrophobins genes from *H. irregulare***

**Number of hydrophobins**	**Recombination events/sites**	**Tajima’s D test**	**Non-synonymous substitutions (dN)**	**Synonymous substitutions (dS)**	**dN/dS ratio**
13	1	−1.03462	189.97	65.03	2.92

### Microarray analysis of hydrophobin expression in *H. annosum* s.s

To investigate the transcriptional regulation of hydrophobins in *H. annosum* s.s. during growth in culture filtrate from *P. gigantea* and to see the biocontrol potentials of the culture filtrate from *P. gigantea*, microarray analysis was conducted. Also microarray analysis of hydrophobins from *H. annosum* s.s. was carried out during saprotrophic growth on wood. Sixteen transcripts of class I hydrophobin-encoding genes with different regulatory patterns were observed (Table 3). Among the 16 hydrophobin transcripts identified, high expression of transcript number 5 was observed mostly during saprotrophic growth of *H. annosum* s.s. on bark, sapwood and heartwood*.* However, transcript number 11 was highly up-regulated during growth on sapwood and heartwood but showed low expression during saprotrophic growth on bark. The abundance of transcript number 9 was observed during saprotrophic growth on bark, sapwood and heartwood as well as during growth on culture filtrate produced by *P. gigantea.*

**Table 3:** **Microarray expression**^**a **^**analysis of hydrophobin encoding genes from *H. annosum *** **s.s. during growth on wood and growth on culture filtrate from *P. gigantea***

**No**	**Transcript ID**	**Pg/Ha fold changes**^**b**^	**P-value**	**Bark/control fold changes**^**b**^	**P-value**	**Sapwood/control fold changes**^**b**^	**P-value**	**Heartwood/control fold changes**^**b**^	**P-value**
1	jgi|Hetan1|181099|AOL_estExt_fgenesh3_kg.C_90057	0.82	0.40	0.50	0.02	0.52	0.02	0.34	0.02
2	jgi|Hetan1|28315|gw1.9.943.1	4.50	0.43	1.21	1.00	0.23	0.52	1.92	0.72
3	jgi|Hetan1|33224|estExt_Genewise1.C_30567	0.59	0.42	1.01	0.98	1.09	0.99	1.89	0.28
4	jgi|Hetan1|46054|e_gw1.3.836.1	1.85	0.90	3.22	0.59	2.39	0.61	1.33	1.00
5	jgi|Hetan1|65822|estExt_Genewise1Plus.C_90176	0.47	0.42	73.57	0.09	17.33	0.21	55.44	0.11
6	jgi|Hetan1|104521|Genemark.5594_g	2.45	0.60	0.32	0.52	0.32	0.51	0.32	0.53
7	jgi|Hetan1|105914|Genemark.6987_g	3.26	0.42	1.22	0.92	4.88	0.06	1.31	0.80
8	jgi|Hetan1|148119|estExt_fgenesh3_kg.C_90130	0.94	1.00	0.88	0.69	0.91	0.87	1.17	0.72
9	jgi|Hetan1|156762|estExt_fgenesh2_pm.C_90143	7.14	0.40	185.8	0.05	202.8	0.04	191.7	0.05
10	jgi|Hetan1|181098|AOL_estExt_fgenesh3_kg.C_90132	0.82	0.40	0.67	0.02	0.78	0.16	0.81	0.21
11	jgi|Hetan1|181114|AOL_EuGene18000072	1.00	1.00	1.46	0.78	33.23	0.02	284.3	0.00
12	jgi|Hetan1|104521|Genemark.5594_g	2.44	0.59	0.32	0.52	0.32	0.51	0.32	0.52
13	jgi|Hetan1|181117|AOL_EuGene16000006	5.39	0.58	0.45	0.83	0.63	0.98	1.07	1.00
14	jgi|Hetan1|156763|estExt_fgenesh2_pm.C_90144	3.89	0.28	0.93	0.62	0.60	0,10	0.73	0.16
15	jgi|Hetan1|17575|gw1.11.458.1	0.38	0.61	0.80	1.00	0.02	0.09	0.73	0.95
16	jgi|Hetan1|181100|AOL_e_gw1.9.435.1	0.11	0.45	0.59	0.07	0.64	0.08	0.58	0.09

^a^= Expression data normalized to liquid Hagem media.

^b^= Fold changes calculated as the expression value of the experimental sample over the control.

## Discussion

Hydrophobins are surface-active proteins with diverse roles in fungal life cycle [9,11-15]. Hydrophobins are grouped into class I and class II based on the following criteria: minimum of eight cysteines arranged in a conserved pattern, solubility of the formed aggregates, hydropathy pattern and the differences in the spacing of amino acids between the cysteine residues [1,36]. Although we did not carry out solubility test in this study, a clear separation of the hydrophobins screened in this study into class I and II was observed. Although *H. irregulare* and *P. brevispora* have different ecological lifestyles, the domain structure of hydrophobins from these two fungi did not differ considerably, an indication that the differences in nutritional lifestyle could not be explained by their domain structures. Separation of hydrophobins into various classes based on hydropathy pattern has been reported in other studies [37].

The most prominent feature of the phylogenetic grouping of hydrophobins from all the fungal species is the separation of the sequences along the two classes of hydrophobins (Additional file 2: Figure S1). Similar results based on phylogenetic grouping of Class I and II into the two major fungal taxa have been reported [3,37]. Some fungal species such as *M. grisea*, *T. virens*, *N. discreta*, *N. crassa* and *N. tetrasperma* have both class I and class II proteins which were resolved into distinct clades (Additional file 2: Figure S1). Similar findings have been reported in other studies where both class I and class II hydrophobins were identified in a single fungal species [1,38]. Class I hydrophobins were common among the members of the basidiomycetes, while both class I and II were present in the ascomycetes. This is in line with findings from other studies [3,37]. The absence of class II proteins in basidiomycetes may indicate that only the class I proteins may be important in basidiomycetes for fruit body formation. However, despite the high level of sequence conservation between the hydrophobins from *P. brevispora* and *H. annosum* s.l., the two fungi appeared to be phylogenetically distant from each other (Additional file 2: Figure S1 and Additional file 3: Figure S2).

Numerous clusters of hydrophobins were found in our analysis; paralogous genes with high bootstrap support were also nested together in the generated trees. The presence of these clusters provides additional evidence in support of our hypothesis about important role of gene duplication in the evolution of hydrophobin-encoding genes in fungi. Paralogous sequences of hydrophobins from *P. brevispora* clustered together and they appeared to be phylogenetically closer to hydrophobins from *W. cocos*, *B. adusta* and *F. mediterranea* (Additional file 2: Figure S1 and Additional file 3: Figure S2). Sequences of hydrophobins from *H. irregulare* were not clearly resolved into distinct clades. It was interesting to note that hydrophobins from *A. delicata*, a basidiomycete were closer relatives to the class I proteins from ascomycetes than members of the same class basidiomycetes (Additional file 2: Figure S1). Hydrophobins of *A. delicata* differ from hydrophobins of most remaining basidiomycetes in our analysis by having shorter region between the conserved cysteines C3 and C4 (26–29 amino residues versus 32–33). This structural feature might influence the results of the phylogenetic analysis. At the same time, *Auricularia* is the most basal member of basidiomycetes in our analysis, and it is possible that hydrophobins of *Auricularia* retained the similarity to the hypothetical ‘ancestral’ type of hydrophobins that were present in the common ancestor of ascomycetes and basidiomycetes, whereas in evolutionary more advanced species of basidiomycetes they are more diverged from that type, e.g. by having longer stretch of amino acids between C3 and C4 positions. The phylogenetic relationships between class I hydrophobins from ascomycetes and the same class of proteins from the basidiomycetes may suggest that the ancestor of these genes may have been formed very early during evolution. Furthermore, it is possible that the gene products may have a role in the life style or ecology of the fungal species. Based on this result, a complete survey of genome sizes and copy numbers of hydrophobin-encoding genes was made on several fungal species with varied ecological strategies (Additional file 4: Figure S3 and Additional file 5: Figure S4). A considerable variation in the number of hydrophobin-encoding genes exists in all the fungi screened, ranging from 1 in *A. alcalophilum* to 40 in *T. versicolor*. In *H. irregulare* 13 hydrophobins have previously been reported [35]. In our analysis, 16 functional class I proteins and 8 putative pseudogene were identified. This difference in the number of hydrophobin-encoding genes observed in our study could be a result of automatic annotation problems. However, our microarray studies identified only the 16 functional proteins. In *P. brevispora,* 26 class I hydrophobin-encoding genes have been identified. Accumulation of several copies of duplicate hydrophobin genes from a single copy of ancestral sequence may have resulted in the variation in numbers of hydrophobin genes in the different fungi. An interesting finding in this work is the absence of hydrophobins among the members of hemiascomycetous yeasts, an indication that species like *S. cerevisiae, Pichia stipitis, Hansenula polymorpha* and *Wickerhamomyces anomalus* with yeast-like or monocentric growth pattern (non-filamentous growth pattern) may not need hydrophobins. In addition, the complete absence of hydrophobins in the *Pucciniales* may suggest that hydrophobin genes may have undergone relaxed selection after evolution. It is also possible that hydrophobins were completely lost during evolution. Among the basidiomycetes screened in this study, a significant expansion of the gene was observed (*P =* 0.002) whereas ascomycetes appeared to have witnessed a massive contraction of the gene during evolution. The expansion of hydrophobin-encoding genes among the members of Basidiomycota may not be unconnected with fruit body formation [9]. It is also possible that the increase in the number of hydrophobin-encoding genes may have been positively selected for in basidiomycetes, but not in ascomycetes. Relationship between the number of hydrophobin-encoding genes and ecological strategy (pathogenic and non-pathogenic life style) was further investigated. There was a well-supported link between the number of hydrophobin genes and ecological strategy with the non-pathogenic fungi having higher numbers of hydrophobins than the pathogenic ones (*P =* 0.0001), this may suggest that although hydrophobins may be needed in fungal pathogenesis [11-15], higher numbers of the gene may have more ecological role in non-pathogenic conditions such as symbiosis [21], mycorrhiza formation [22] and interspecific fungi-fungi interactions [20,23], fruit body formation [39] and emergence of hyphal structures [18-20]. This result is in contrast with other studies that have shown massive expansion of gene families in pathogens as compared with non-pathogenic relatives [31,32]. Differences between ecological strategies and genome size were also tested. Genome size had no significant effect on ecological strategy (*P =* 0.1).

Evolutionary forces operating at a genomic scale may have some influence on gene family expansion or contractions, implying that the fungal species with larger genome sizes may have a correspondingly higher number of hydrophobins. However, there was no significant relationship between genome size and the number of hydrophobin-encoding genes in this study. To understand the evolutionary forces driving hydrophobin gene family evolution in *H. irregulare*, a selection test was carried out. A high dN/dS ratio (> 1) was observed in *H. irregulare* hydrophobins, an indication that hydrophobin genes may be under positive selection. It is also possible that the evolution of these genes could be a result of recombination and duplication events. Evidence of duplication events have been reported in hydrophobins from *P. gigantea*[20,23] and *H. irregulare*[12]. These finding may therefore suggest that this group of proteins may have evolved through the so called birth and death model [14]. In the birth and death model, new gene copies evolve through duplications followed by diversification due to accumulation of spontaneous mutations; new duplicates with vital functions are retained in the genome while those with deleterious effects are purged from the genome through purifying selection. We identified 16 transcripts of class I hydrophobin encoding genes that were differentially regulated during growth on bark, heartwood and sapwood as well as during growth on culture filtrate produced by *P. gigantea*. Furthermore, the differences in expression pattern of these class I proteins during saprotrophic growth may suggest that different hydrophobin genes are employed by *H. annosum* s.s. during growth on diverse wood components. In addition, the low transcript abundance observed during growth in the submerged medium containing culture filtrate from *P. gigantea* may suggest that the induction of these genes could be inhibited due to the presence of the secreted molecules from the biocontrol agent (*P. gigantea*)*. P. gigantea* is the biological control fungus for the control of *H. annosum* s.l. infection. It is possible that one of the mechanisms for action of the biocontrol fungus against the pathogen is by repression of genes such as hydrophobins through its secreted molecules or metabolites. Other studies have reported the upregulation of hydrophobin encoding genes at the zone of interaction between the biocontrol fungus and the pathogen [20,23]. Due to lack of protein structure of hydrophobins from *H. annosum* s.l., hydrophobins from selected fungal species were modeled alongside hydrophobins from *P. gigantea*. The models revealed the surface patches of hydrophobic residues (Figure 3), which are possibly important in the formation of amphiphilic membranes as reported earlier [5]. The conserved cysteine residues confirm probably a preserved structural feature of the protein.

## Conclusions

We have surveyed the distribution and evolution of hydrophobin genes in *P. brevispora* and *H. irregulare* as well as in other fungi. We have also examined the relationships between the number of hydrophobin-encoding genes and ecological strategy in the examined fungal species. From our results, hydrophobin genes have witnessed a considerable expansion in *P. brevispora* as well as in other basidiomycetes while contraction of the same gene family has occurred in the ascomycetes. In addition, although different numbers of hydrophobins have been reported in *P. brevispora, H. annosum* s.l. and other fungal species, each gene has different regulatory pattern in the pathogenic fungus (*H. annosum* s.s.) during growth on wood and in culture filtrate produced by the antagonistic *P. gigantea*. The results from this study have also given some insights on some of the factors underlying the ecological habits of the fungi screened in this study.

## Methods

### Gene mining

Annotated sequences of hydrophobins from *H. irregulare*[12] and *P. brevispora* were used as queries to search for other hydrophobin sequences of some randomly selected fungal species, using BlastP [40]. Blast searches were performed at the fungal genomic platform of the Joint Genome Institute (JGI) (http://www.jgi.doe.gov/). The identified ORFs were used as queries to search for all possible hydrophobin proteins in the selected fungal species. Repeated blast searches were carried out until no more hydrophobin proteins were found. Sequences with E-values below 10^-5^ were selected for further analysis. Due to the reasonably lower number of hits generated by the blast results, filtered model was further used as a criterion to download all the hydrophobin proteins of the selected fungal taxa. Sequences without N- or C-terminal parts were corrected. Also sequences with regions of unspliced introns were corrected and aligned using MUSCLE alignment tool implemented in Molecular Evolutionary Genetic Analysis software (MEGA 5.0). MUSCLE was used because it gives a better accuracy and is computationally stronger than CLUSTAL alignment [41]. The aligned protein sequences were viewed with the Biological sequence alignment editor (Bioedit), Windows 95/98/NT/2 K/XP [42]. Alignments were curated manually, and all ambiguous positions were removed. The sequences were further filtered to remove pseudogenes, or sequences shorter than 50 residues in length, or missing the hydrophobin domains as defined by the InterproScan Tool [43] or having a different gene ontology (GO) from hydrophobins and realigned for further analysis. The signal peptides were predicted using SignalP 3.0 software. The protein sequences were classified into class I and class II using Kyte-Doolittle hydropathy plot, Version 2.0u66 in Windows 9.0 [44] and published consensus sequence information for class I [33] and class II [14] respectively.

### Species tree reconstruction

A total of 335 protein sequences from 41 fungal species (Additional file 1: Table S1) were used for the analysis. The protein sequences were further divided into the two major fungal taxa, basidiomycetes and ascomycetes. Species-based trees were reconstructed for both groups using the Neighbor-joining (NJ) method in MEGA. The evolutionary distances were calculated using the Jones Taylor–Thorton (JTT) + gamma matrix-based [34] taking into account rate heterogeneity among sites. The rate variation among sites was calculated with a gamma distribution with a default parameter of 5. The reliability of internal branches was evaluated using 1000 bootstrap replications [45].

### Species ecological strategy, genome size and hydrophobin distribution

Information on the ecological strategy of each fungal species was obtained from already published data. In parallel, a complete survey of hydrophobin distribution and genome sizes of all the fungi species used in the study was obtained from JGI (http://www.jgi.doe.gov/) (Additional file 1: Table S1). The fungal species were divided into basidiomycetes and ascomycetes, and were further subdivided according to their ecological strategies, pathogens and non-pathogens (Additional file 1: Table S1). The selected fungal species (Additional file 1: Table S1) were analyzed for gene contraction through gene loss and gene expansion through duplication. The relationship between ecological strategy and the copy number of hydrophobin-encoding genes within the selected set of species was also examined (Additional file 1: Table S1). Further analysis to determine the relationship between genome size and hydrophobin gene distribution was evaluated among the fungi in this group.

### Microarray expression analysis of hydrophobin genes from the pathogenic fungus, *H. annosum* s.s. during growth on culture filtrate of *P. gigantea* and saprotrophic growth on pine wood

#### Saprotrophic growth of *H. annosum s.s.* on wood bark, sapwood and heartwood

Wood discs from Scots Pine (*Pinus sylvestris*) were separated into bark, sapwood and heartwood. Each wood component was grounded into small particles of sizes 0.5-1 mm for 15 min at 590 rpm using a ball grinding mill (Fritsch Pulverisette, Germany) and 8 g of each wood material was weighed in a flask and autoclaved for 20 min. The wood tissues were allowed to cool for 20 min, 8 ml sterile low nitrogen medium (NH_4_NO_3_ 0.6 g/L, K_2_HPO_4_ 0.4 g/L, KH_2_PO_4_ 0.5 g/L, MgSO_4_·7H_2_0 0.4 g/L) was added to each flask followed by the addition of 8 ml of sterile distilled water ensuring that comparable moisture levels was maintained. This was followed by inoculation with three pieces of 1 × 1 cm agar plugs of malt extract agar containing *H. annosum* s.s. hyphae (isolate FP5, obtained from Kari Korhonen, Finish Forest Research Institute (METLA), Vantaa Finland). The plugs were put into each flask, mixed gently to allow the agar plugs to be covered by the wood tissues. Cultures were incubated at 20°C and harvested after 3 months. Harvested mycelia and wood tissues were frozen in liquid nitrogen and stored at −80°C until RNA extraction. There were 3 biological replicates for each sample.

#### Growth of *H. annosum* s.s. in culture filtrates of *P. gigantea*

Three Erlenmayer flask (300 ml) containing liquid malt extract (100 ml) each, were inoculated with 3 mm agar plugs of the commercial isolate of *P. gigantea* (Rotstop®), courtesy of Kari Korhonen (Finish Forest Research Institute (METLA), Vantaa Finland)*.* Cultures were incubated at 20°C for 10 days. The cultures were filtered to get rid of the fungal mycelia using sterile flask and filter paper. The liquid filtrate was incubated overnight at 70°C to kill any remaining particle of *P. gigantea* mycelia. Freshly growing agar plugs (3 mm) of *H. annosum* s.s. were inoculated in each flask and cultures were incubated at 20°C. In parallel, fresh liquid medium of malt extract were inoculated with 3 mm freshly growing agar plugs from *H. annosum* s.s. to act as positive control and incubated at 20°C. Cultures were harvested after 10 days post inoculation (d.p.i) and the harvested mycelia were frozen in liquid nitrogen until further processing. There were 3 biological replicates for each experiment.

#### RNA processing and microarray

RNA was extracted from triplicate cultures of each sample using the method by Chang [46] with some modifications. RNA was purified by using the RNeasy® MinElute Cleanup kit (QIAGEN) according to the protocol. RNA integrity was assessed with RNA 6000 Nano kit using an Agilent Bioanalyzer (Agilent, CA). RNA concentration was measured using NanoDrop ND-1000 Spectrophotometer and the purity of the samples was estimated by the OD ratios (A_260_/A_280_, ranging within 1.8–2.2). The RNA samples were DNase treated to remove any potential DNA contamination using DNaseI according to the recommendations from the manufacturer (Fermentas, Canada). cDNA was synthesized by using the TransPlex® Complete Whole Transcriptome Amplification Kit according to the manufacturer’s protocol (SIGMA). Microarray analysis was carried out using the Nimblegene protocol (http://www.nimblegen.com/).

### Statistical analysis

Pearson’s partial correlation coefficient was used to test if the number of hydrophobins in *P. brevispora* or *H. irregulare* and hydrophobins from other fungal species correlate with their genome sizes. General linear model (GLM) procedure in SAS was used to test the hypothesis that the number of hydrophobin-encoding genes and genome size differed between basidiomycetes and ascomycetes, and between pathogens and non-phytopathogens. To understand the evolutionary forces driving hydrophobin gene evolution in *H. irregulare,* recombinations events were tested using the method described in [47]; also the ratio of non-synonymous substitution per site (dN) to synonymous substitution per site (dS) in 13 paralogous sequences of hydrophobin-encoding genes was calculated. Tajima’s *D* test statistics [48] was also applied on the hydrophobin-encoding genes from *H. irregulare*. For the microarray data, the mean expression and fold changes were calculated with FDR (Benjamini Hochberg) multiple testing corrections using ArraySTAR software (3801 Regent Street Madison, WI53705, USA). Student t-test was also used to determine differences in mean between samples.

### Homology modeling of hydrophobins

Similar sequences to the hydrophobins from *P. gigantea* and *H. irregulare* as well as hydrophobins from *Verticillium dahliae, T. terrestris*, *U. maydis* and *L. bicolor* were located in the entries of Protein Data Bank (PDB) using PSI-BLAST search [49] and aligned using CLUSTAL W [50]. The best predictions were selected as the templates and these structures were obtained from the PDB [51], then superimposed and compared with the programs LSQMAN [52] and O [53]. The best pair-wise alignments with the relevant parts of the structures were used to generate homology models of the hydrophobins from *P. gigantea*, *H. irregulare*, *V. dahliae*, *T. terrestris*, *U. maydis* and *L. bicolor* with thioredoxin reductase from *Drosophila melanogaster* (PDB entry 3DGH; identity 35%), transcriptional regulator BT_p548217 from *Bacteroides thetaiotaomicron* (PDB entry 2K9Q; identity 35%), Hydrophobin from *Hypocrea jecorina* (PDB entry 2FZ6; identity 44%) [7], hydrophobin from *N. crassa* (PDB entry 2K6A [54]; identity 48%), Dsl1p subunit from *Saccharomyces cerevisiae* (PDB entry 3ETU [55]; identity 31%) and glyceraldehyde-3-phosphate dehydrogenase from *Trypanosoma cruzi* (PDB entry 3DMT [56]; identity 30%) structures respectively as templates in the program SOD [53]. The models were adjusted in O, using rotamers that would improve packing in the interior of the protein, and accounting for insertions and deletions in loop regions. The models are available upon request from the authors. The figure was prepared using http://www.pymol.org.

## Availability of supporting data

The microarray data obtained in this work were deposited at the Gene Expression Omnibus (GEO, http://www.ncbi.nlm.nih.gov/geo/) database (accession number GSE39805 (http://www.ncbi.nlm.nih.gov/geo/query/acc.cgi?acc=GSE39805) and GSE41301 (http://www.ncbi.nlm.nih.gov/geo/query/acc.cgi?acc=GSE41301). The alignments of hydrophobin sequences used for the phylogenetic reconstructions were deposited at the TreeBASE database (http://www.treebase.org/) under accession number 14520 (http://purl.org/phylo/treebase/phylows/study/TB2:S14520).

## Competing interests

The authors declare that they have no competing interests.

## Authors’ contributions

ACM designed the experiments, carried out the phylogenetic analysis, bioinformatic and statistical analysis, did part of the microarray work and drafted the manuscript. AK contributed in the phylogenetic analysis and manuscript preparation. HC contributed in the microarray work on saprotrophic growth of the fungus. WU did the work on protein modeling. FOA conceived the study, directed the work and contributed in writing the manuscript. All authors read and approved the final manuscript.

## Supplementary Material

Additional file 1: Table S1 Fungal species screened in this study, their ecological strategies, genome sizes and numbers of predicted hydrophobin-encoding genes. Click here for file

Additional file 2: Figure S1 The phylogenetic tree of hydrophobins from a representative set of basidiomycetes and ascomycetes. Neighbor-Joining tree showing the phylogenetic relationships between selected fungal hydrophobins. Bootstrap support values above 30 (in percent) are indicated next to the branches. Clade **A** = class I hydrophobins from both ascomycetes and basidiomycetes, clade **B** (blue) = class II hydrophobins from ascomycetes. Subclade C = Class I proteins from ascomycetes (magenta) and basidiomycetes (red) *A. delicata* marked with grey, *M. grisea*, *N. tetrasperma*, *N. discreta*, *N. crassa* and *T. virens* have both classes I and II proteins and are marked with black circle at the tip of the branches. *T. terrestris* nested with class I proteins from ascomycetes and is marked with black triangle at the tip of the branch. Subclade D (green) = class I proteins from basidiomycetes of the Order Polyporales, Subclasses E (pink), F (black) and G (purple) = Class I hydrophobins from basidiomycetes of different systematic positions and ecological preferences. Other unmarked subclades are shown in black, *U. maydis* marked with black square. Following abbreviations are used to indicate the fungal species: |Lacbi2|, *Laccaria bicolor*; |Hetan2|, *Heterobasidion irregulare*; |Phlbr1|, *Phlebia brevispora*; |Bjead1|, *Bjerkandera adusta*; |Gansp1|, *Ganoderma* sp.; |Phchr1|, *Phanerochaete chrysosporium;* |Serla_varsha1|, *Serpula lacrymans*; |Wolco1|, *Wolfiporia cocos*; |Cersu1|, *Ceriporiopsis subvermispora*; |Copci1|, *Coprinopsis cinerea*; |Schco2|*, Schizophyllum commune*, |Fomme1|, *Fomitiporia mediterranea*; |Fompi3|, *Fomitopsis pinicola*; |Punst1|, *Punctularia strigosozonata*; |Trave1|, *Trametes versicolor*; |Conpu1|, *Coniophora puteana*; |Glotr11|, *Gloeophyllum trabeum*; |Pospl1|, *Postia placenta*; |Thite2|, *Thielavia terrestri*s; |Ustma1|, *Ustilago maydis*; |Acral2|, *Acremonium alcalophilum*; |Aspca3|,*Aspergillus carbonarius*; |Aspcl1|, *Aspergillus clavatus*; |Aspnid1|, *Aspergillus nidulans*; |Dotse1|, *Dothistroma septosporum*; *|*Lepmu1*|, Leptosphaeria maculans*; |Triat2|, *Trichoderma atroviride*; |TriviGv|, *Trichoderma virens*; |Aurde1|, *Auricularia delicata*; |Dacsp1|, *Dacryopinax* sp.; |Altbr1|, *Alternaria brassicicola*; |Triha1|, *Trichoderma harzianum*; |Pench1|, *Penicillium chrysogenum*; |Neudi1|, *Neurospora discreta*; |Fusgr1|, *Fusarium graminearum*; |Fusox1|, *Fusarium oxysporum*; |Maggr1|, *Magnaporthe grisea*; |Neucr1|, *Neurospora crassa*; |Neute_mat_a1|, *Neurospora tetrasperma*; |Verda1|, *Verticillium dahliae*. Click here for file

Additional file 3: Figure S2 Phylogenetic tree showing the relationships between hydrophobins from a representative set of basidiomycetes. The tree was inferred using the Neighbor-Joining method. The optimal tree with the sum of branch length = 76.88625866 is shown. The percentage of replicate trees in which the associated taxa clustered together in the bootstrap test (1000 replicates) are shown above the branches. The evolutionary distances were computed using the JTT matrix-based method and are in the units of the number of amino acid substitutions per site. The analysis involved 281 amino acid sequences. All ambiguous positions were removed for each sequence pair. There were a total of 216 positions in the final dataset. All the sequences of hydrophobins are from class I proteins except the sequence from *U. maydis* which has some deviations from the general consensus of class I proteins. Seven major clades **H**, **I**, **J**, **K**, **M**, **N** and **O**. Clades **L** and **P** are smaller clades. Clades **H**, **I** and **J** = Class I proteins from basidiomycetes of the Order Polyporales, Clade **K** = Class I hydrophobins from basidiomycetes of different orders (Agaricales, Polyporales, Russulales, Corticiales and Boletales), Clades **L** and **P** = Hydrophobins from basidiomycetes of different orders and lifestyles, **M** = Mostly sequences of hydrophobins from *S. commune* and *A. delicata*., **N** = Hydrophobins sequences from Agaricales, Boletales and Dacrymycetales, **O** = Hydrophobins from brown rotting fungi (Boletales). The following abbreviations are used to indicate the fungal species: |Lacbi2|, *Laccaria bicolor*; |Hetan2|, *Heterobasidion irregulare*; |Phlbr1|, *Phlebia brevispora;* |Bjead1|, *Bjerkandera adusta;* |Gansp1|, *Ganoderma* sp.; |Phchr1|, *Phanerochaete chrysosporium*; |Serla_varsha1|, *Serpula lacrymans*; |Wolco1|, *Wolfiporia cocos*; |Cersu1|, *Ceriporiopsis subvermispora*; |Copci1|, *Coprinopsis cinerea*; |Schco2|*, Schizophyllum commune*; |Fomme1|, *Fomitiporia mediterranea*; |Fompi3|, *Fomitopsis pinicola*; |Punst1|, *Punctularia strigosozonata*; |Trave1|, *Trametes versicolor*; |Conpu1|, *Coniophora puteana*; |Glotr11|, *Gloeophyllum trabeum*; |Pospl1|, *Postia placenta.* Click here for file

Additional file 4: Figure S3 Genome size and hydrophobin-encoding genes copy number in basidiomycetes. Comparison of the genome sizes (in Mbp) and the copy number of hydrophobin-encoding genes in the species of basidiomycetes analyzed in this study. Click here for file

Additional file 5: Figure S4 Genome size and hydrophobin-encoding genes copy number in ascomycetes. Click here for file

Additional file 6: Table S2 Inventory of hydrophobin encoding genes in *P. brevispora.* Click here for file

Additional file 7: Table S3 Inventory of hydrophobin encoding genes in *H. irregulare.* Click here for file
