# Supplementary material for: Evolutionary analysis of hydrophobin gene family in two wood-degrading basidiomycetes, Phlebia brevispora and Heterobasidion annosum s.l
Source: BMC Evol Biol. 2013 Nov 4;13:240. doi: 10.1186/1471-2148-13-240 (PMC3879219; doi:10.1186/1471-2148-13-240)
Supplement: Additional file 1: Table S1 — Fungal species screened in this study, their ecological strategies, genome sizes and numbers of predicted hydrophobin-encoding genes. [file 1471-2148-13-240-S1.docx]

**Supplementary Table 1** Fungal species screened in this study, their ecological strategies, genome sizes and numbers of predicted hydrophobin-encoding genes

| Phylum | Species | Lifestyle | Ecological strategy^a^ | Genome size (MB) | Gene number^b,c^ | Reference |
| --- | --- | --- | --- | --- | --- | --- |
| Basidiomycota | *Auricularia delicata* | Saprotroph/ White rot | NP | 74.92 | 5 | http://genome.jgi.doe.gov/Aurde1/Aurde1.home.html |
| Basidiomycota | *Coprinopsis cinerea* | Saprotroph | NP | 37.5 | 33 | [1] |
| Basidiomycota | *Pleurotus ostreatus* | Saprotroph/ White rot | NP | 35.6 | 25 | http://genome.jgi.doe.gov/PleosPC9_1/PleosPC9_1.home.html |
| Basidiomycota | *Phlebia brevispora* | Saprotroph/ White rot | NP | 49.96 | 26 | http://genome.jgi.doe.gov/Phlbr1/Phlbr1.home.html |
| Basidiomycota | *Agaricus bisporus* | Saprotroph | NP | 30.2 | 22 | http://genome.jgi.doe.gov/Agabi_varbisH97_2/Agabi_varbisH97_2.home.html |
| Basidiomycota | *Laccaria bicolor* | Mycorrhiza/  Symbiont | NP | 60.71 | 13 | [2] |
| Basidiomycota | *Ceriporiopsis subvermispora* | Saprotroph/  White-rot | NP | 39.0 | 25 | http://genome.jgi.doe.gov/Cersu1/Cersu1.home.html |
| Basidiomycota | *Serpula lacrymans* | Saprotroph/  Brown rot | NP | 42.4 | 17 | [3] |
| Basidiomycota | *Punctulariastrigosozonata* | White rot/saprotroph | NP | 34.17 | 27 | http://genome.jgi.doe.gov/Punst1/Punst1.home.html |
| Basidiomycota | *Dichomitus squalens* | Saprotroph / White rot | NP | 42.75 | 21 | http://genome.jgi.doe.gov/Dicsq1/Dicsq1.home.html |
| Basidiomycota | *Fomitiporia*  *mediterranea* | Pathogen / White rot | PP | 63.35 | 19 | http://genome.jgi.doe.gov/Fomme1/Fomme1.home.html |
| Basidiomycota | *Trametes versicolor* | Saprotroph / White rot | NP | 44.79 | 40 | http://genome.jgi.doe.gov/Trave1/Trave1.home.html |
| Basidiomycota | *Heterobasidion*  *irregulare* | Pathogen / White rot | PP | 33.7 | 13 | [4] |
| Basidiomycota | *Fomitopsis*  *pinicola* | Saprotroph / Brown rot | PP;NP | 46.30 | 7 | http://genome.jgi.doe.gov/Fompi1/Fompi1.home.html |
| Basidiomycota | *Phanerochaete*  *carnosa* | Saprotroph / White-rot | PP | 46.29 | 15 | http://genome.jgi.doe.gov/Phaca1/Phaca1.home.html |
| Basidiomycota | *Postia placenta* | Saprotroph / Brown rot | NP | 33.0 | 3 | [5] |
| Basidiomycota | *Ustilago*  *maydis* | Pathogen | PP | 20.0 | 2 | [6] |
| Basidiomycota | *Stereum*  *hirsutum* | Saprotroph/White rot | PP | 46.51 | 0 | http://genome.jgi.doe.gov/Stehi1/Stehi1.home.html |
| Basidiomycota | *Tremella*  *mesenterica* | Saprotroph / White rot | NP | 28.6 | 0 | http://genome.jgi.doe.gov/Treme1/Treme1.info.html |
| Basidiomycota | *Cryptococcus neoformans* | Pathogen | HP | 19.5 | 0 | [7] |
| Basidiomycota | *Melampsora*  *larici-populina* | Biotrophic/path-ogenic | PP | 101.1 | 0 | http://genome.jgi.doe.gov/Mellp1/Mellp1.home.html |
| Basidiomycota | *Sporobolomyces roseus* | Saprotroph | NP | 21.2 | 0 | http://genome.jgi.doe.gov/Sporo1/Sporo1.info.html |
| Basidiomycota | *Rhodotorula*  *graminis* | Saprotroph | NP | 21.0 | 0 | http://genome.jgi.doe.gov/Rhoba1_1/Rhoba1_1.info.html |
| Basidiomycota | *Puccinia*  *graminis* | Biotroph | PP | 88.84 | 0 | http://genome.jgi.doe.gov/Pucgr1/Pucgr1.home.html |
| Ascomycota | *Neurospora*  *tetrasperma* | Saprotroph | NP | 39.1 | 2 | http://genome.jgi.doe.gov/Neute_matA2/Neute_matA2.info.html |
| Ascomycota | *Neurospora*  *discreta* | Saprotroph | NP | 37.3 | 2 | http://genome.jgi.doe.gov/Neudi1/Neudi1.info.html |
| Ascomycota | *Neurospora*  *crassa* | Saprotroph | NP | 41.04 | 2 | [8] |
| Ascomycota | *Acremonium*  *alcalophilum* | Saprotroph | NP | 54.42 | 1 | http://genome.jgi.doe.gov/Acral2/Acral2.home.html |
| Ascomycota | *Saccharomyces cerevisiae* | Saprotroph | NP | 12.07 | 0 | [9] |
| Ascomycota | *Pichia stipitis* | Saprotroph | NP | 15.4 | 0 | http://genome.jgi.doe.gov/Picst3/Picst3.info.html |
| Ascomycota | *Hansenula polymorpha* | Saprotroph | NP | 8.97 | 0 | http://genome.jgi.doe.gov/Hanpo2/Hanpo2.home.html |
| Ascomycota | *Wickerhamomycesanomalus* | Saprotroph | NP | 14.15 | 0 | http://genome.jgi.doe.gov/Wican1/Wican1.info.html |
| Ascomycota | *Aspergillus*  *nidulans* | Saprotroph | NP | 30.48 | 3 | http://genome.jgi.doe.gov/Aspnid1/Aspnid1.home.html |
| Ascomycota | *Chaetomium globosum* | Saprotroph / pathogen | HP; NP | 34.9 | 0 | http://genome.jgi.doe.gov/Chagl_1/Chagl_1.home.html |
| Ascomycota | *Pichia membranifaciens* | Saprotroph | NP | 11.58 | 0 | http://genome.jgi.doe.gov/Picme2/Picme2.home.html |
| Ascomycota | *Thielavia terrestris* | Saprotroph | NP | 36.91 | 1 | http://genome.jgi.doe.gov/Thite2/Thite2.home.html |
| Zygomycota/ Mycoromycotina | *Mucor circinelloides* | Saprotroph | NP | 36.5 | 0 | http://genome.jgi-psf.org/Mucci1/Mucci1.home.html |
| Ascomycota | *Aspergillus*  *carbonarius* | Saprotroph | NP | 36.3 | 4 | http://genome.jgi.doe.gov/Aspca3/Aspca3.home.html |
| Ascomycota | *Aspergillus*  *niger* | Saprotroph | NP | 34.85 | 4 | [10] |
| Ascomycota | *Aspergillus*  *aculeatus* | Saprotroph | NP | 35.4 | 3 | http://genome.jgi.doe.gov/Aspac1/Aspac1.home.html |
| Zygomycota / Mucoromycotina | *Phycomyces blakesleeanus* | Saprotroph | NP | 55.9 | 0 | http://genome.jgi-psf.org/Phybl1/Phybl1.home.html |
| Ascomycota | *Magnaporthe grisea* | Hemibiotroph/  phytopathogens | PP | 41.70 | 4 | [11] |
| Ascomycota | *Trichoderma virens* | mycoparasite | NP | 39.0 | 11 | [12] |
| Ascomycota | *Trichoderma atroviride* | mycoparasite | NP | 36.1 | 11 | [12] |
| Ascomycota | *Fusarium oxysporum* | Saprotroph/ pathogen | PP | 61.36 | 3 | http://genome.jgi-psf.org/Fusox1/Fusox1.home.html |
| Ascomycota | *Fusarium graminearum* | Pathogen | PP | 36.49 | 2 | http://genome.jgi-psf.org/Fusgr1/Fusgr1.home.html |
| Ascomycota | *Alternaria brassicicola* | Pathogen/  saprotroph | PP | 30.3 | 2 | <http://genome.jgi> psf.org/Altbr1/Altbr1.info.html |
| Ascomycota | *Dothistroma septosporum* | Pathogen/  saprotroph | PP | 30.21 | 3 | http://genomeportal.jgi-psf.org/Dotse1/Dotse1.home.html |
| Ascomycota | *Leptosphaeria maculans* | Pathogen/  saprotroph | PP | 44.89 | 1 | http://genomeportal.jgi-psf.org/Lepmu1/Lepmu1.home.html |
| Ascomycota | *Batrachochytrium*  *dendrobatidis* | Pathogen | AP | 24.3 | 0 | http://genome.jgi-psf.org/Batde5/Batde5.home.html |

^a^= (NP= Non phytopathogen, PP= Phytopathogen, AP= Animal pathogen, HP= Human pathogen)

^b^ = Number of predicted hydrophobin-encoding genes

^c^= Number of hydrophobins from unpublished references are based on automated results from the sequence assembly

**References**

1. Stajich JE, Wilke SK, Ahren D, Au CH, Birren BW, Borodovsky M, Burns C, Canbaeck B, Casselton LA, Cheng CK, Deng J, Dietrich FS, Fargo DC, Farman ML, Gathman AC, Goldberg J, Guigo R, Hoegger PJ, Pukkila PJ: **Insights into evolution of multicellular fungi from the assembled chromosomes of the mushroom *Coprinopsis cinerea* (*Coprinopsis cinereus*)**. *Proc Natl Acad Sci. U.S.A.* 2010, **107**:11889–11894.

2. Martin F, Aerts A, Ahren D, Brun A, Danchin EGJ, Duchaussoy F, Gibon J, Kohler A, Lindquist E, Pereda V, Salamov A, Shapiro HJ, Wuyts J, Blaudez D, Buee M, Brokstein P, Canbaeck B, Cohen D Grigoriev IV: The genome of *Laccaria bicolor* provides insights into mycorrhizal symbiosis. *Nature* 2008, **452**:88–92.

3. Eastwood DC, Floudas D, Binder M, Majcherczyk A, Schneider P, Aerts A, Asiegbu FO Baker SE, Barry K, Bendiksby M, Blumentritt M, Coutinho PM, Cullen D, et al: **Plant cell wall-decomposing machinery underlies the functional diversity of forest Fungi.** *Sci* 2011, **333**:762–765.

4. Olson Å, Aerts A, Asiegbu F, Belbahri L, Bouzid O, Broberg A, Canbäck B, Coutinho PM, Cullen D, Dalman K, Deflorio G, van Diepen LTA, Dunand C, Duplessis S, Durling M, Gonthier P, Grimwood J, Fossdal CG, Hansson D, *et al*: **Insight into trade-off between wood decay and parasitism from the genome of a fungal forest pathogen.** *New Phytol* 2012, **194:**1001–1013.

5. Martinez D, Challacombe J, Motgenstern I, *et al*: **Genome, transcriptome, and secretome analysis of wood decay fungus** ***Postia placenta*** **supports unique mechanisms of lignocellulose conversion.** *Proc Nat Acad Sci U S A* 2009, **106**(6)**:**1954–1959.

6. Jörg K, Regine K, Michael B, Li-Jun M, Thomas B, Barry JS, Flora B, James WK, Scott EG, Olaf M, *et al*: **Insights from the genome of the biotrophic fungal plant pathogen,** ***Ustilago maydis*.** *Nature* 2006, **444:**97–101.

7. Loftus BJ, Fung E, Roncaglia P, Rowley D, Amedeo P, Bruno D, Vamathevan J, Miranda M, Anderson IJ, Fraser JA, Allen JE, Bosdet IE, Brent MR, Chiu R, Doering TL, Donlin MJ, *et al*: **The genome of the basidiomycetous yeast and human pathogen** ***Cryptococcus neoformans*.** *Sci* 2005, **307:**1321–1324.

8. James EG, Sarah EC, Katherine AB, Eric US, Nick DR, *et al*: **The genome sequence of the filamentous fungus** ***Neurospora crassa*.** *Nature* 2003, **422:**859–868.

9. Kellis M, Patterson N, Endrizzi M, Birren B, Lander ES: **Sequencing and comparison of yeast species to identify genes and regulatory elements.** *Nature* 2003, **423:**241–254.

10. Herman JP, Johannes HW, David BA, Paul SD, Gerald H, Peter JS, Geoffrey T, *et al*: **Genome sequencing and analysis of the versatile cell factory** ***Aspergillus niger*** **CBS 513.88.** *Nat Biotechnol* 2007, **25:**221–231.

11. Dean RA, Talbot NJ, Ebbole DJ, Farman ML, Mitchell TK, Orbach MJ, Thon M, Kulkarni R, Xu JR, Pan H, Read ND, Lee YH, *et al*: **The genome sequence of the rice blast fungus** ***Magnaporthe grisea*.** *Nature* 2005, **434**(7036)**:**980–986.

12. Kubicek CP, Herrera-Estrella A, Seidl-Seiboth V, Martinez DA, Druzhinina IS, Thon M, Zeilinger S, Casas-Flores S, Horwitz BA, Mukherjee PK, Mukherjee M, *et al*: **Comparative genome sequence analysis underscores mycoparasitism as the ancestral life style of** ***Trichoderma*.** *Gen Biol* 2011, **12:**R40.
