# Supplementary figures and images for: Evolutionary analysis of hydrophobin gene family in two wood-degrading basidiomycetes, Phlebia brevispora and Heterobasidion annosum s.l
Source: BMC Evol Biol. 2013 Nov 4;13:240. doi: 10.1186/1471-2148-13-240 (PMC3879219; doi:10.1186/1471-2148-13-240)

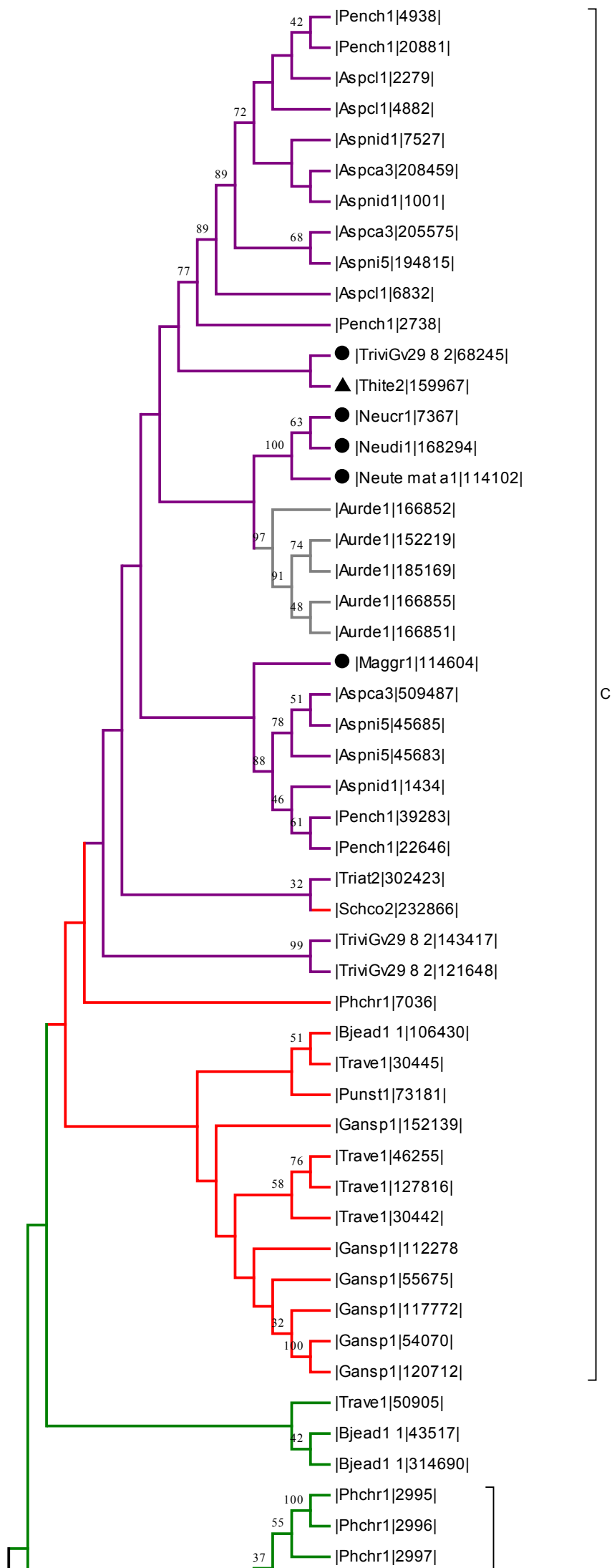

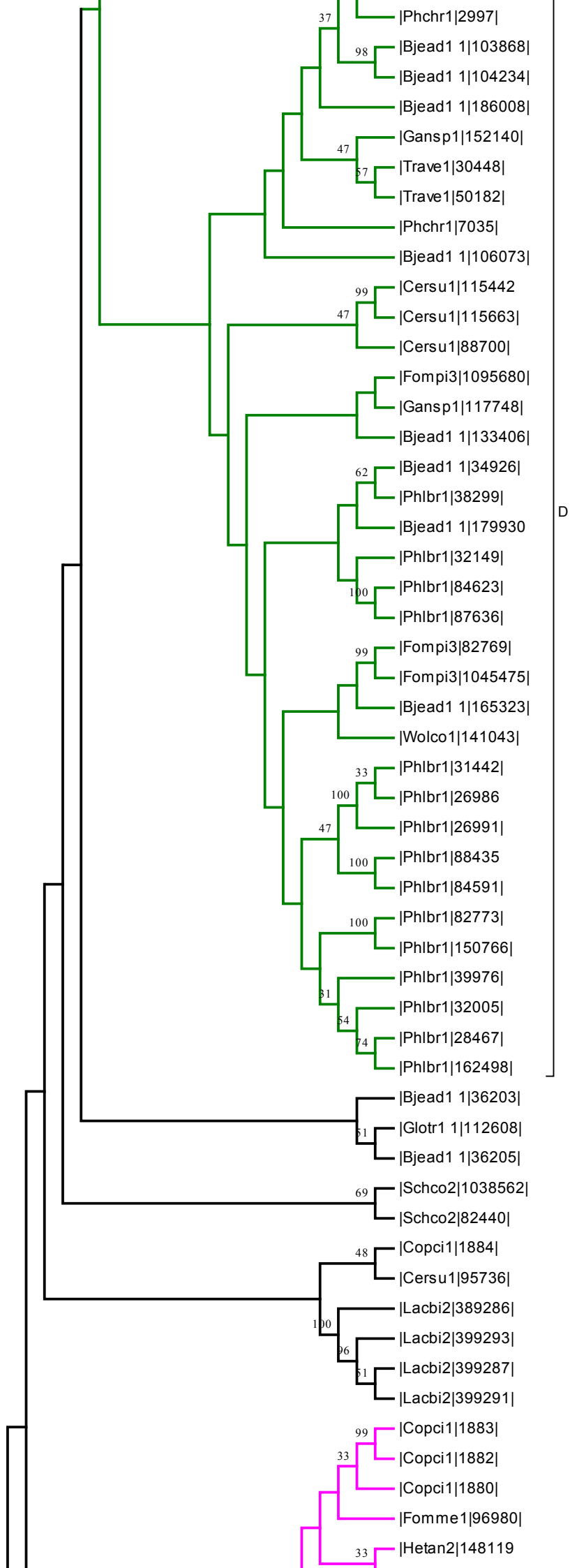

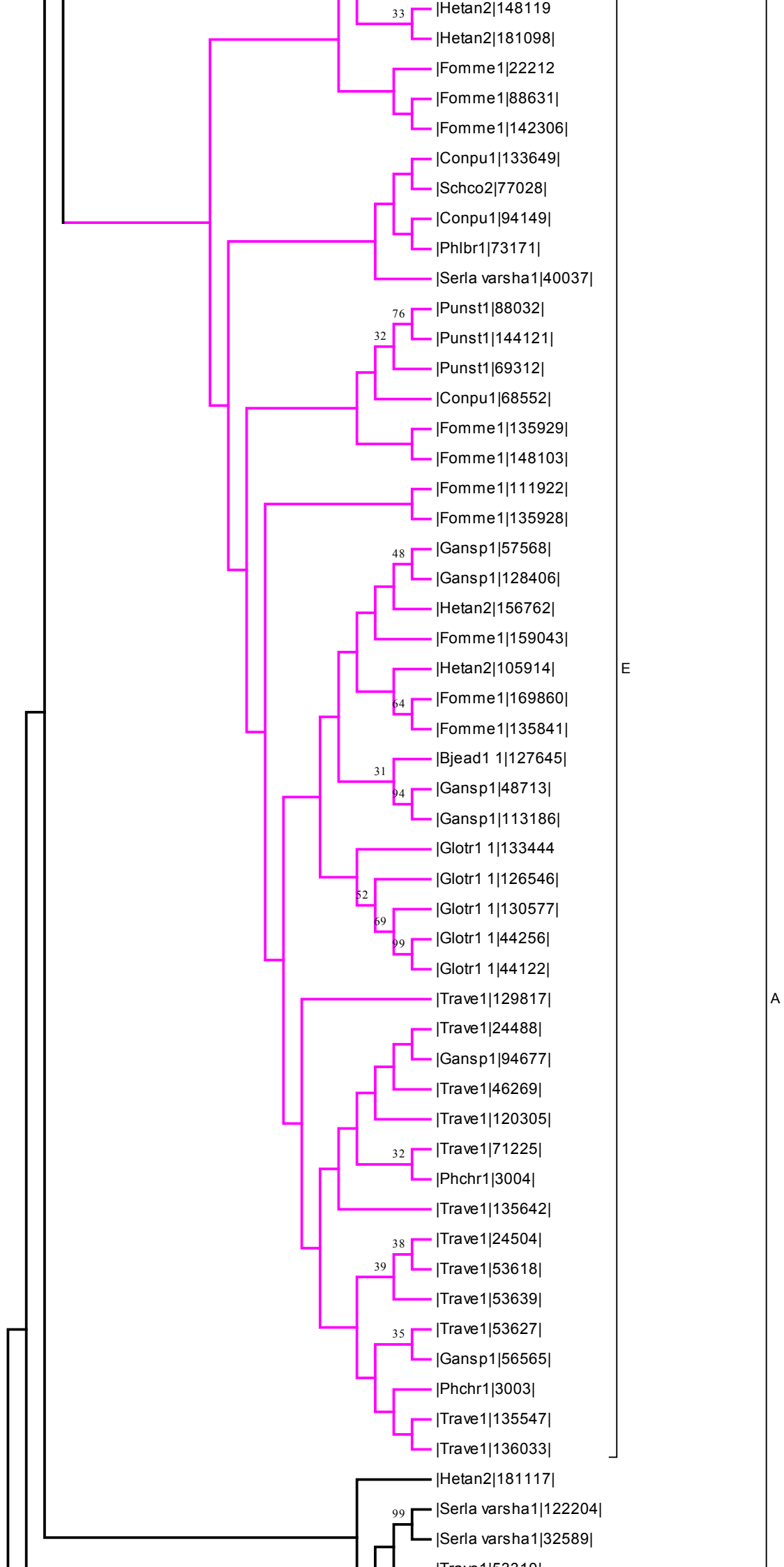

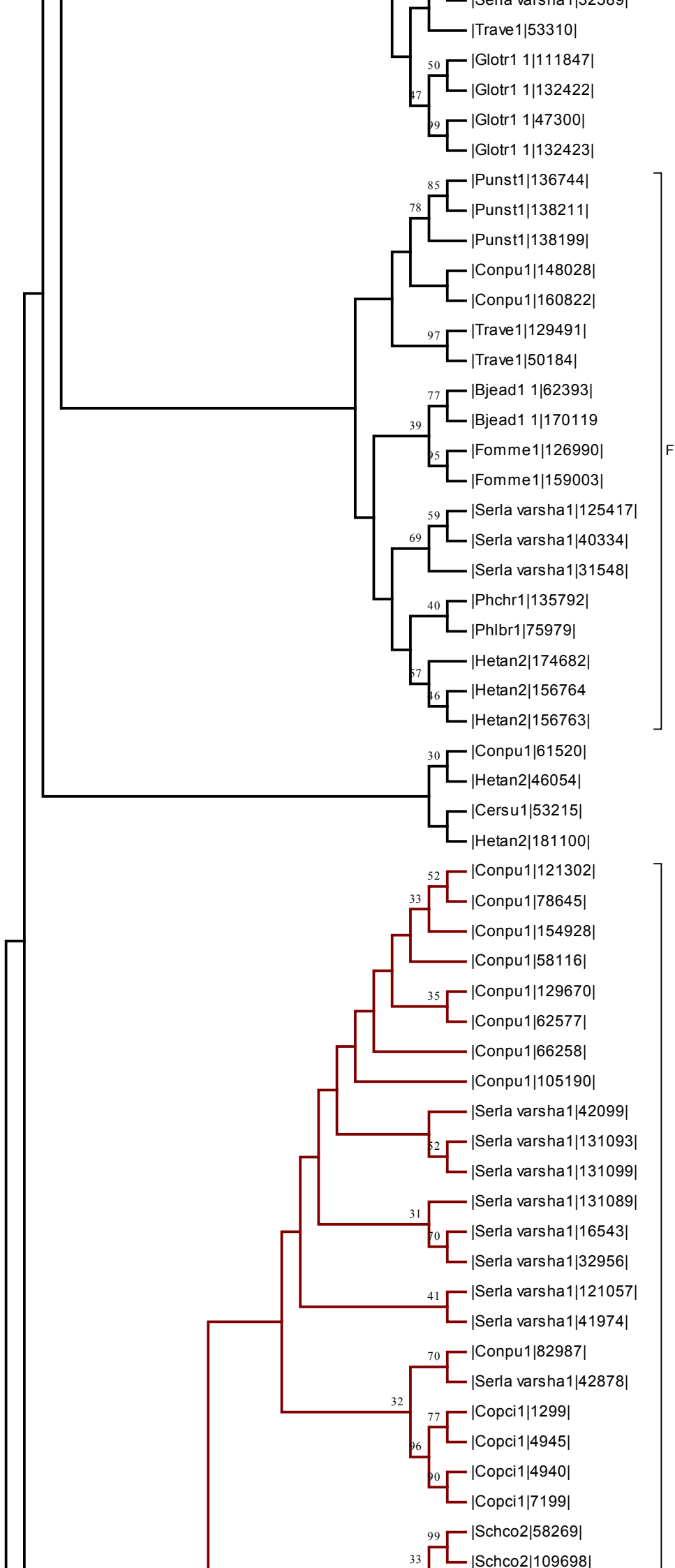

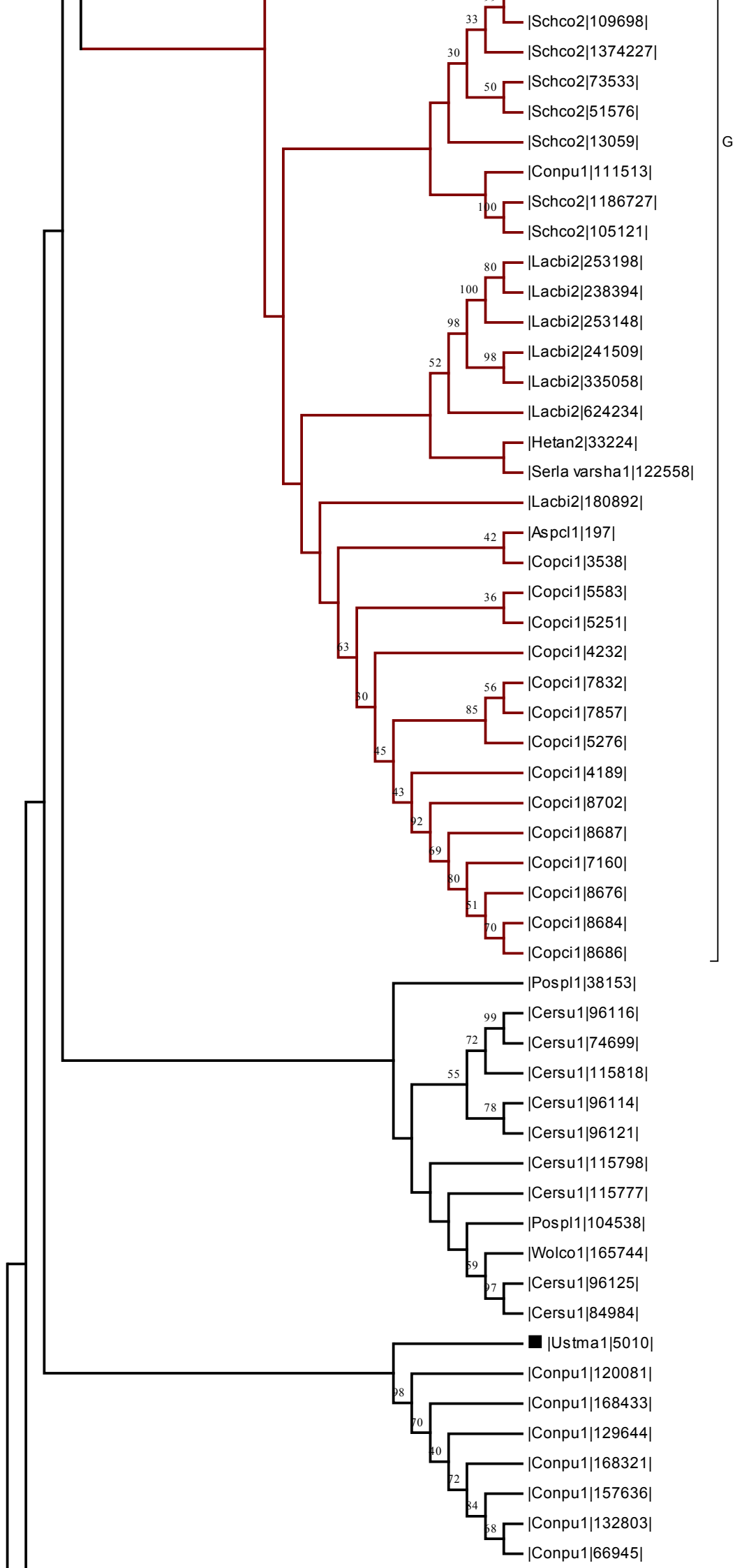

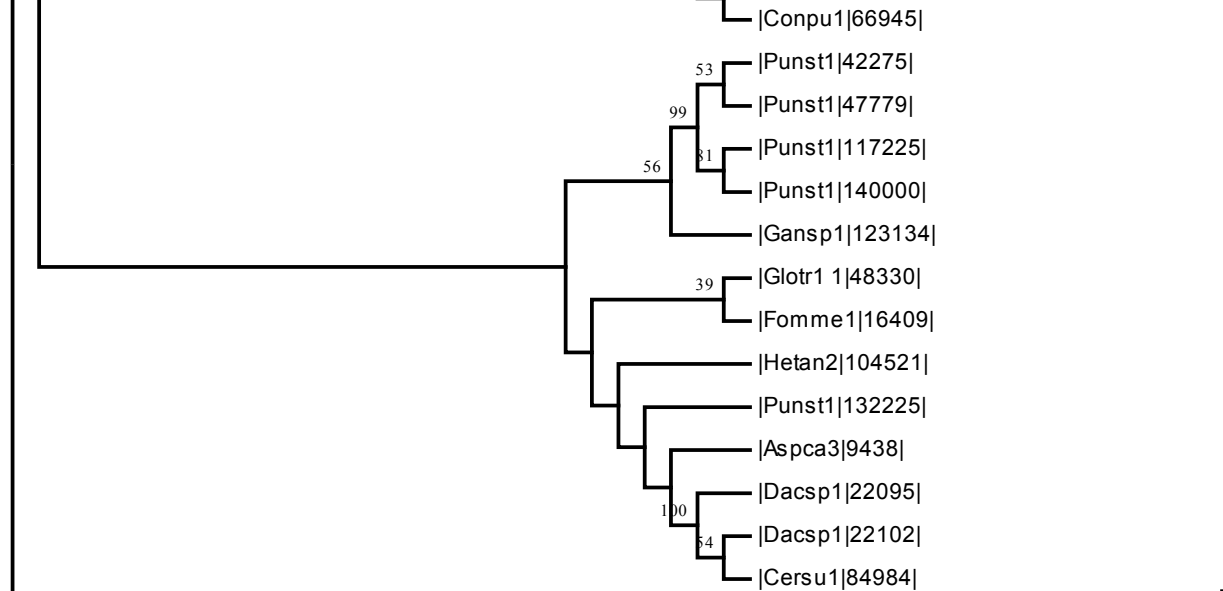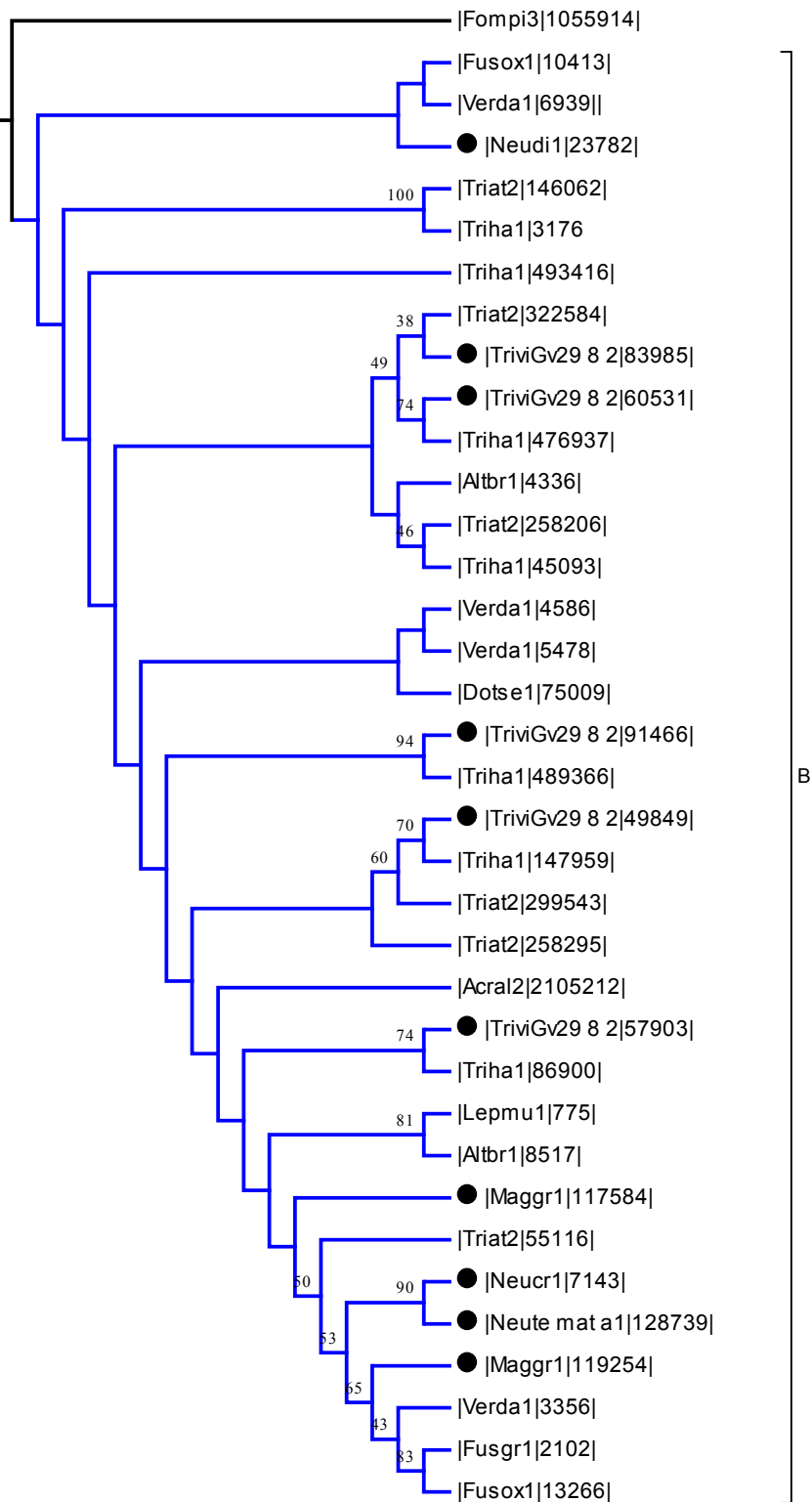

Supplement: Additional file 2: Figure S1 — The phylogenetic tree of hydrophobins from a representative set of basidiomycetes and ascomycetes. Neighbor-Joining tree showing the phylogenetic relationships between selected fungal hydrophobins. Bootstrap support values above 30 (in percent) are indicated next to the branches. Clade A = class I hydrophobins from both ascomycetes and basidiomycetes, clade B (blue) = class II hydrophobins from ascomycetes. Subclade C = Class I proteins from ascomycetes (magenta) and basidiomycetes (red) A. delicata marked with grey, M. grisea, N. tetrasperma, N. discreta, N. crassa and T. virens have both classes I and II proteins and are marked with black circle at the tip of the branches. T. terrestris nested with class I proteins from ascomycetes and is marked with black triangle at the tip of the branch. Subclade D (green) = class I proteins from basidiomycetes of the Order Polyporales, Subclasses E (pink), F (black) and G (purple) = Class I hydrophobins from basidiomycetes of different systematic positions and ecological preferences. Other unmarked subclades are shown in black, U. maydis marked with black square. Following abbreviations are used to indicate the fungal species: |Lacbi2|, Laccaria bicolor; |Hetan2|, Heterobasidion irregulare; |Phlbr1|, Phlebia brevispora; |Bjead1|, Bjerkandera adusta; |Gansp1|, Ganoderma sp.; |Phchr1|, Phanerochaete chrysosporium; |Serla_varsha1|, Serpula lacrymans; |Wolco1|, Wolfiporia cocos; |Cersu1|, Ceriporiopsis subvermispora; |Copci1|, Coprinopsis cinerea; |Schco2|, Schizophyllum commune, |Fomme1|, Fomitiporia mediterranea; |Fompi3|, Fomitopsis pinicola; |Punst1|, Punctularia strigosozonata; |Trave1|, Trametes versicolor; |Conpu1|, Coniophora puteana; |Glotr11|, Gloeophyllum trabeum; |Pospl1|, Postia placenta; |Thite2|, Thielavia terrestris; |Ustma1|, Ustilago maydis; |Acral2|, Acremonium alcalophilum; |Aspca3|,Aspergillus carbonarius; |Aspcl1|, Aspergillus clavatus; |Aspnid1|, Aspergillus nidulans; |Dotse1|, Dothistroma septosporum; |Lepm [file 1471-2148-13-240-S2.pdf]

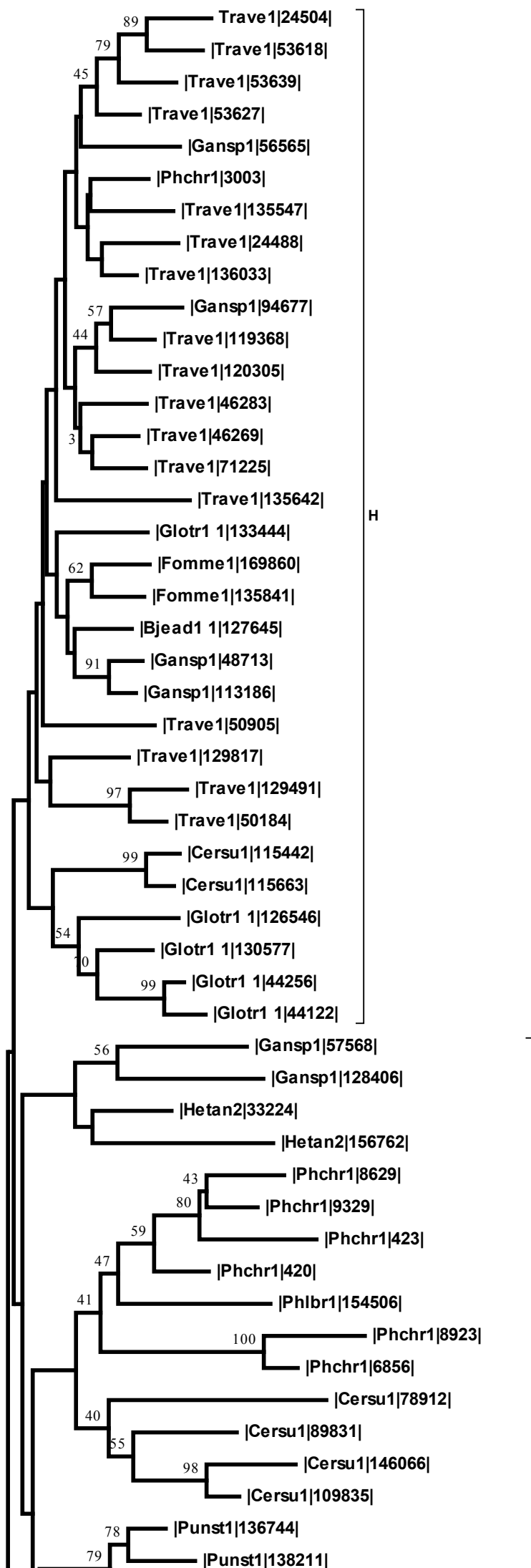

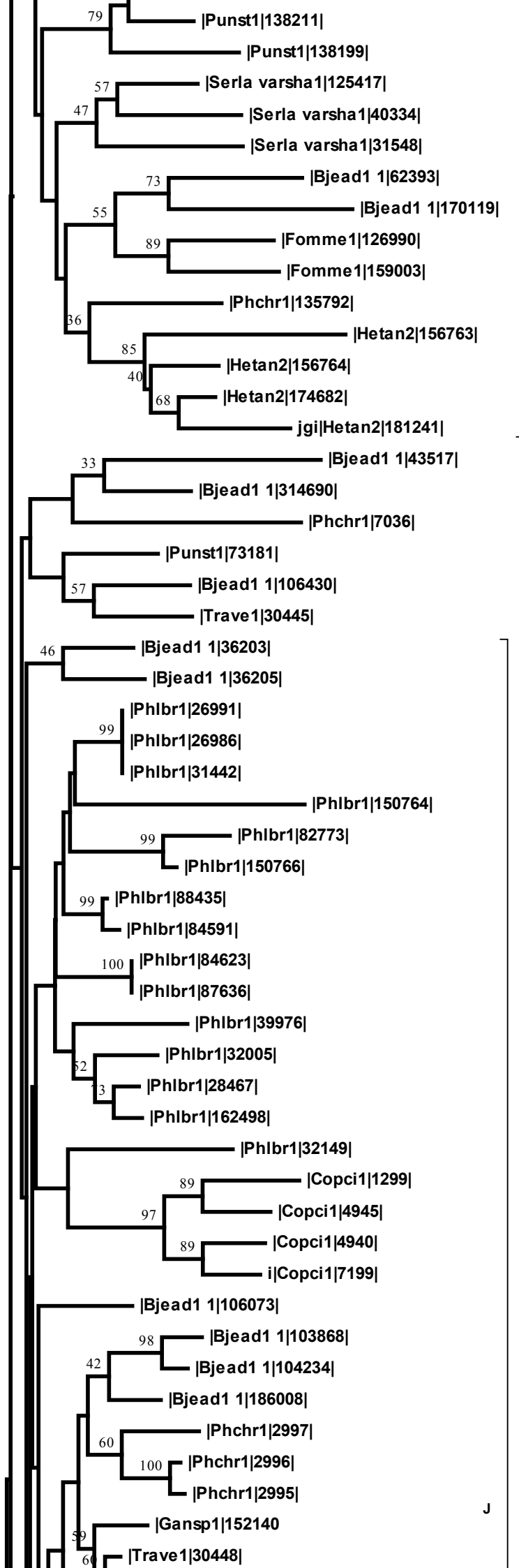

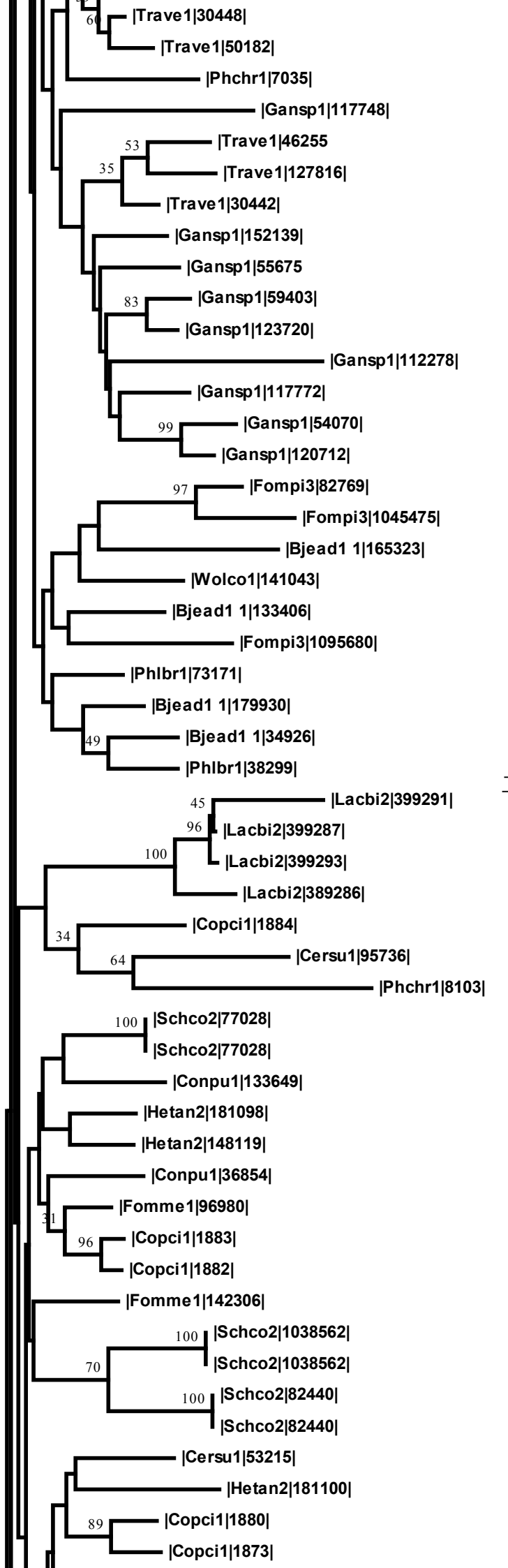

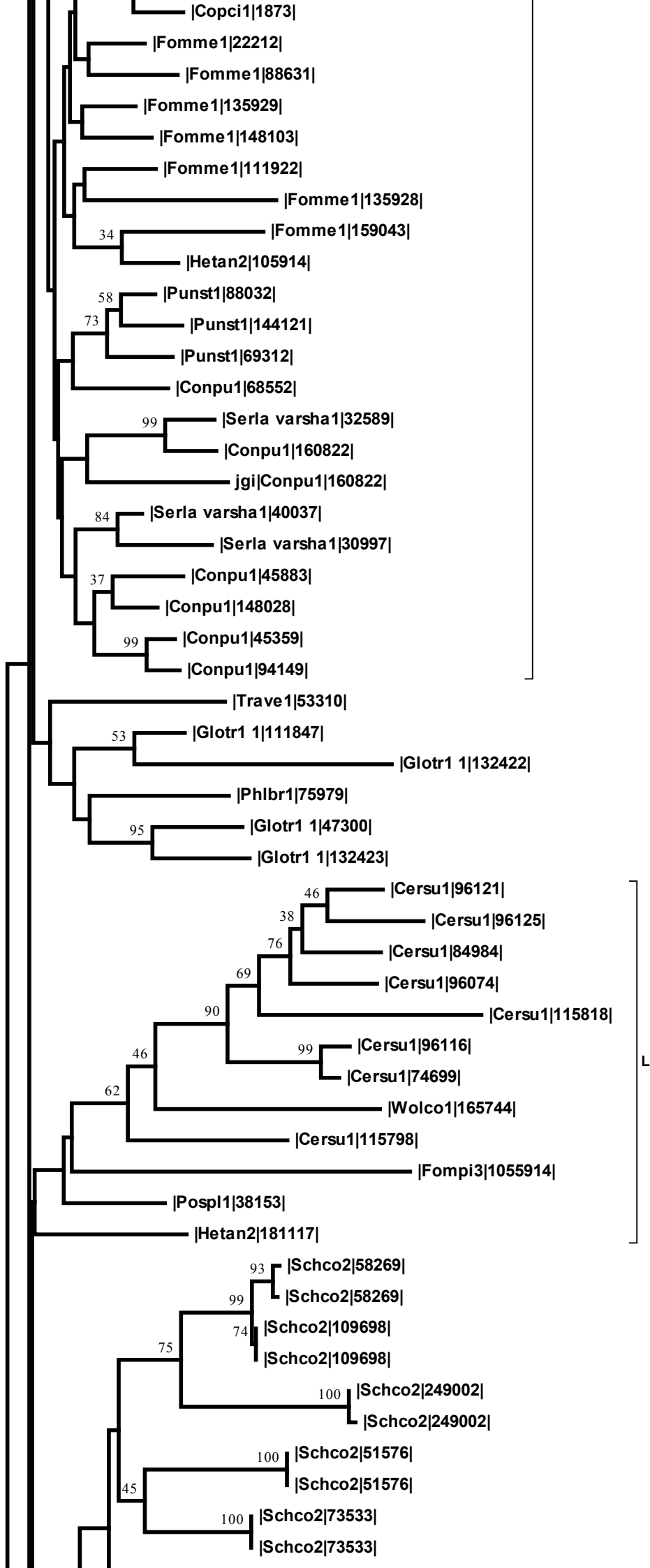

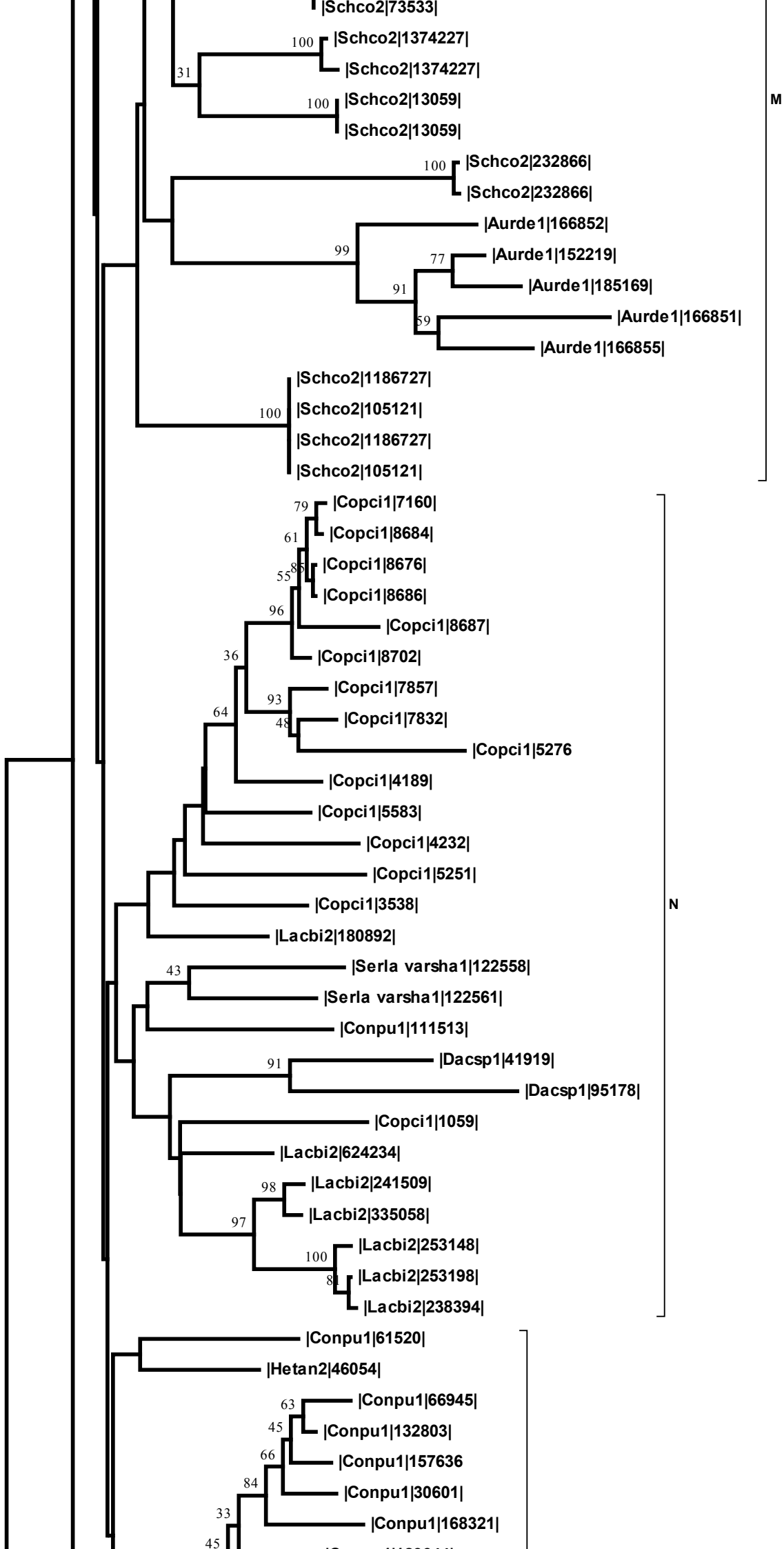

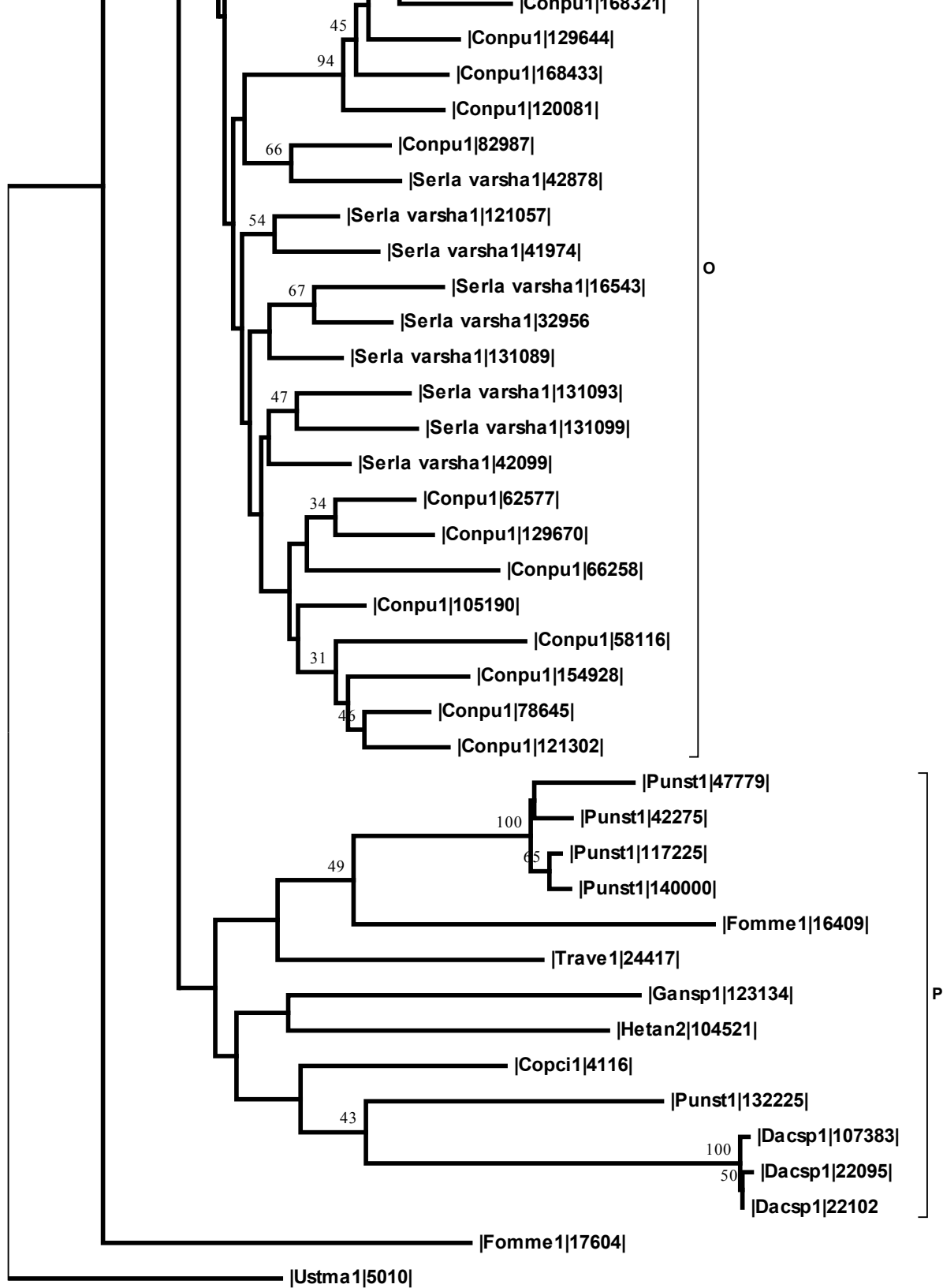

0.2

Supplement: Additional file 3: Figure S2 — Phylogenetic tree showing the relationships between hydrophobins from a representative set of basidiomycetes. The tree was inferred using the Neighbor-Joining method. The optimal tree with the sum of branch length = 76.88625866 is shown. The percentage of replicate trees in which the associated taxa clustered together in the bootstrap test (1000 replicates) are shown above the branches. The evolutionary distances were computed using the JTT matrix-based method and are in the units of the number of amino acid substitutions per site. The analysis involved 281 amino acid sequences. All ambiguous positions were removed for each sequence pair. There were a total of 216 positions in the final dataset. All the sequences of hydrophobins are from class I proteins except the sequence from U. maydis which has some deviations from the general consensus of class I proteins. Seven major clades H, I, J, K, M, N and O. Clades L and P are smaller clades. Clades H, I and J = Class I proteins from basidiomycetes of the Order Polyporales, Clade K = Class I hydrophobins from basidiomycetes of different orders (Agaricales, Polyporales, Russulales, Corticiales and Boletales), Clades L and P = Hydrophobins from basidiomycetes of different orders and lifestyles, M = Mostly sequences of hydrophobins from S. commune and A. delicata., N = Hydrophobins sequences from Agaricales, Boletales and Dacrymycetales, O = Hydrophobins from brown rotting fungi (Boletales). The following abbreviations are used to indicate the fungal species: |Lacbi2|, Laccaria bicolor; |Hetan2|, Heterobasidion irregulare; |Phlbr1|, Phlebia brevispora; |Bjead1|, Bjerkandera adusta; |Gansp1|, Ganoderma sp.; |Phchr1|, Phanerochaete chrysosporium; |Serla_varsha1|, Serpula lacrymans; |Wolco1|, Wolfiporia cocos; |Cersu1|, Ceriporiopsis subvermispora; |Copci1|, Coprinopsis cinerea; |Schco2|, Schizophyllum commune; |Fomme1|, Fomitiporia mediterranea; |Fompi3|, Fomitopsis pinicola; |Punst1|, Punctularia strigosozonata; |Trave1|, Tr [file 1471-2148-13-240-S3.pdf]

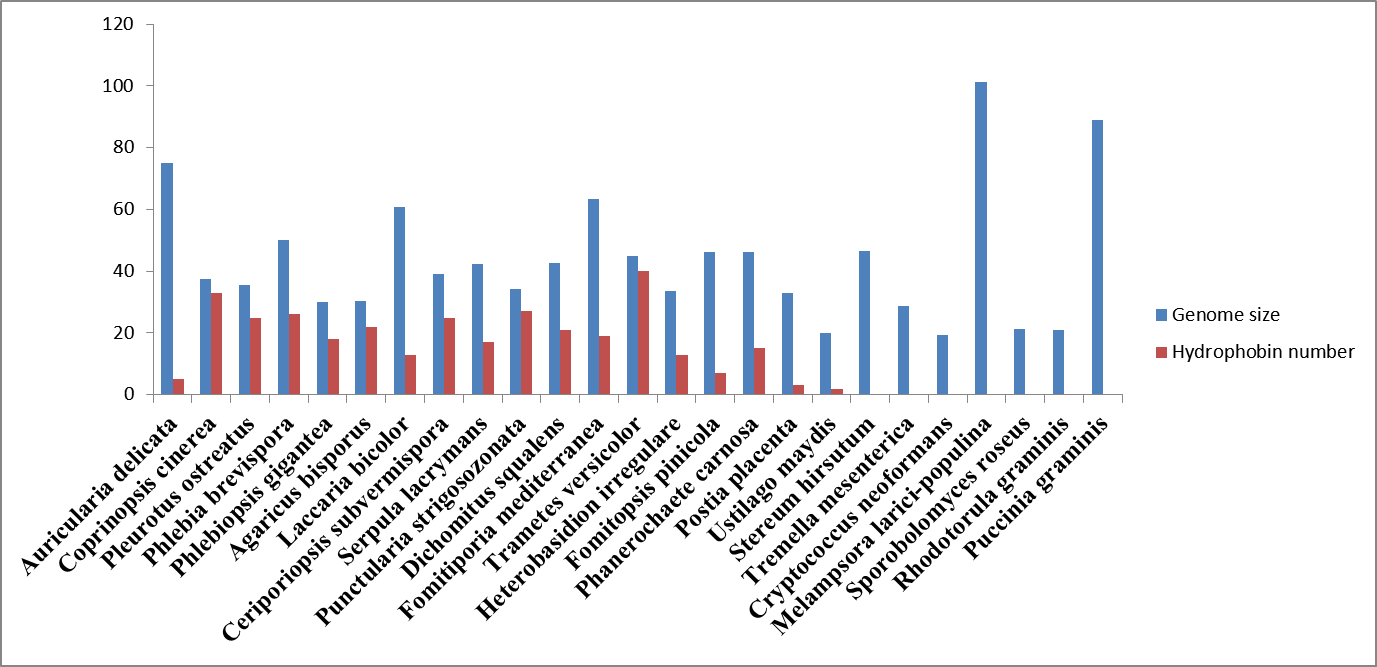

Supplement: Additional file 4: Figure S3 — Genome size and hydrophobin-encoding genes copy number in basidiomycetes. Comparison of the genome sizes (in Mbp) and the copy number of hydrophobin-encoding genes in the species of basidiomycetes analyzed in this study. [file 1471-2148-13-240-S4.png]

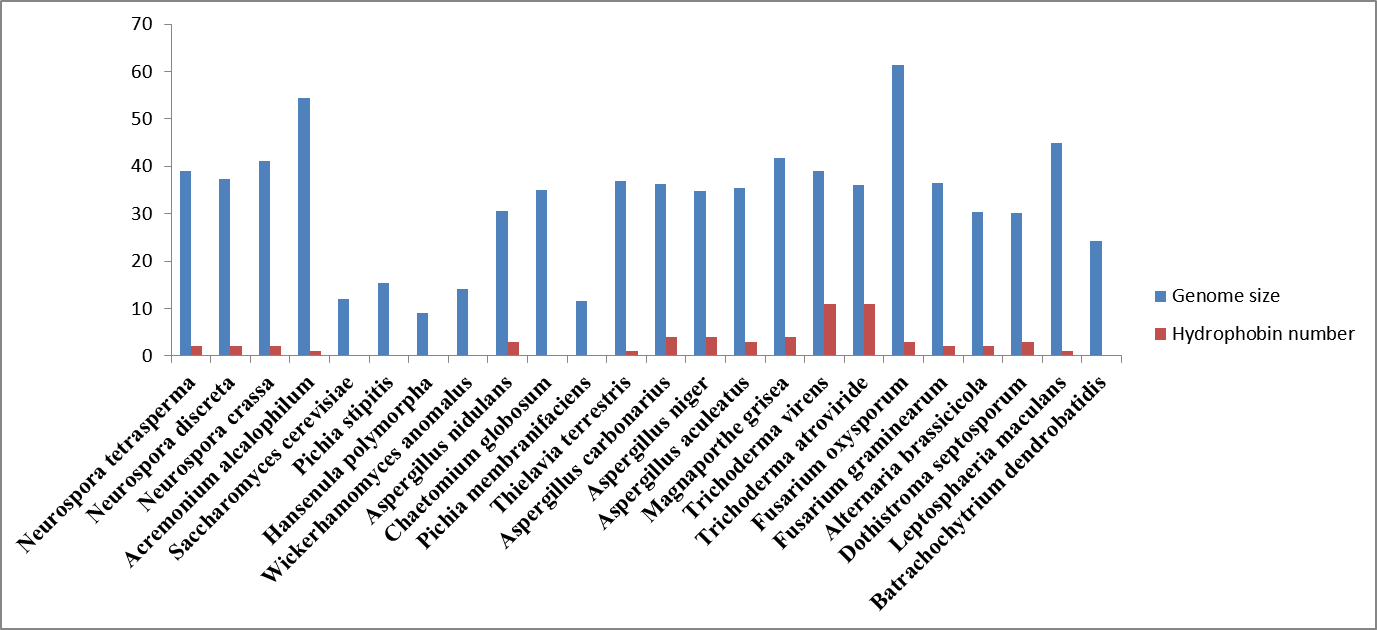

Supplement: Additional file 5: Figure S4 — Genome size and hydrophobin-encoding genes copy number in ascomycetes. [file 1471-2148-13-240-S5.png]
