# Supplementary material for: Evolutionary analysis of hydrophobin gene family in two wood-degrading basidiomycetes, Phlebia brevispora and Heterobasidion annosum s.l
Source: BMC Evol Biol. 2013 Nov 4;13:240. doi: 10.1186/1471-2148-13-240 (PMC3879219; doi:10.1186/1471-2148-13-240)
Supplement: Additional file 6: Table S2 — Inventory of hydrophobin encoding genes in P. brevispora. [file 1471-2148-13-240-S6.docx]

Additional file 6: Table S2: Inventory of *H. annosum* hydrophobins

|  | Model name | Protein ID | Location | Remarks |
| --- | --- | --- | --- | --- |
| 1 | Hetan1.ake_estExt_fgenesh2_pg.C_110275 | \|  \| 181196 \| \| --- \| --- \| | scaffold_04:1864852-1867318 | >jgi\|Hetan2\|181196\|Hetan1.ake_estExt_fgenesh2_pg.C_110275  MLVQLSALSTVSVLGLALLASAAPSEPIPYGSSYGAAAPASASSLAPELYSSAAAPYSQPSPSSEVSAAVPAPYSSESPAPYKEYAPEVYSSAPAPYSSAAPEPSKTPEVYISAPVSYSSPAPVPSRAPEVYSAAPASYSSAAPAVYSSAPAPYSSVVSGAYASVPVSYSSAAPEPSKAPEAYSSAPASYSSAAPAPYKEYAPEVYSSAPAPYSSAAPEVYTSTPVSYSSAAPESSKGPEVYSSAAPAPYKEYAPEVYSSAPAPYSSAAPEVYTSTPVSYSSAAPEPSKGPEVYSSAAPAPYKEYAPEVYSSAPAPYSSVVPEVYSSAPAAYSSVAPGPSKAPEAYSSAPAPYSSAAPTPYKEYAPEVYSSAPAPYSSAALYKAPEVYSSAAPAPYKEYAPEVYSSAPAPYSSVVPEVYSSAPAPSSSPAPEPYKAYVSEVYSSAPASYTSAAPEPYKASPSEVYSSVAMPYSSSTPQVYTSLAPEPYKAYAPEYNSTSAPYAKPTAEAYSSAPAPYSSAPPEVYSPVMASSSAYTIKVYSSANSLPAPAPTPYATWGPKPVAYKRGDYKPGEEPKVYKPEEKRPEVYKPEAKKTEVYPAPAPVYQAIEEHDVKQCNVGELRCCNTVETASATSASKELAVLGLVLQDLNTPIGLNCDALNTGGFGSGSNCAQQPVCCEKNEYGGLAIGCTPVNVVL* Very long protein, major part of the N-terminal is an unknown protein sequence which fused with hydrophobin sequence located at the C-terminal end of the protein sequence. The entire sequence may not be a functional protein |
| 2 | Hetan1.estExt_fgenesh2_pm.C_90144 | 156763 | scaffold_11:965874-967151 | >jgi\|Hetan2\|156763\|Hetan1.estExt_fgenesh2_pm.C_90144  MFARITIVSAVLGLAVLTLAIPSPSGWSDQSLTAECNTGGVQCCNTIESSESESVSSVLAQQGFILQGQNIPIGLNCDTFATDSVGSNCATQPVCCDKTLVGGFGINCNPINFVL*  Normal hydrophobin sequence |
| 3 | Hetan1.estExt_fgenesh2_pm.C_90143 | 156762 | scaffold_11:964656-965384 | >jgi\|Hetan2\|156762\|Hetan1.estExt_fgenesh2_pm.C_90143  MFARLSLLSVLGLVLVAAAHPSPSTKRTGQCNTGKLNCCDTVGKSDDPSISKQLGLLGVVLQGLSVPVGLSCDPIDLIGVGDGSNCAQQPACCTGDVQNGLVNLGCTPINAGL*  Normal hydrophobin sequence |
| 4 | Hetan1.estExt_fgenesh2_pm.C_90145 | \|  \| 156764 \| \| --- \| --- \| | scaffold_11:967244-968020 | >jgi\|Hetan2\|156764\|Hetan1.estExt_fgenesh2_pm.C_90145  MFARFSTLSALSVLAIATMVAAGPSGWHKPEEVVPKHDYKPEYQPEYQPEYKSESKNSLSAQCNTGSVQCCDTVEDASSDNAAKALGLLGLVVQGANVPIGLNCDSIDVLIGVGAGSNCASQPVCCDKTESGLGVGCLPINLAA* Normal hydrophobin sequence |
| 5 | Hatan1.EuGene9000334 | 174682 | scaffold_11:968675-969485 | >jgi\|Hetan2\|174682\|Hetan1.EuGene9000334  MFARFSALSALSILAVAMVASAGPVADYGKSPVAYDPKSDYKPEYAPKEYSKGDYGKDDYGKDNYGKDNYGKDKYGKDVYGKDEYGRDRDGYDDHGRDHYGQDRYGHDDYDPDHRGHGDFDGDRYGQDDYDPDHDDYDNDGYSNDYESQCNSGPIQCCNTVEDASSDAAAKALGLLGLVIQGLNVPIGLDCTPISAGAAFGSGSNCASQP  VCCDKVEEGGIAVGCNPINVVV*  Hydrophobin sequence,reasonable part of the protein sequence are parts of intron. |
| 6 | Hetan1.AOL_EuGene16000006 | \|  \| 181117 \| \| --- \| --- \| | scaffold_09:1661330-1661838 | >jgi\|Hetan2\|181117\|Hetan1.AOL_EuGene16000006  MFARVPTLFLAFFLCLACAASASAHPAAAASRAAAKPSATTKPSQSAAACNTGSIQCCNALQSAGAPGISVILGLLGIKVGDVNAIVGFGCAPITVGGAGAGASCAAQPVCCTGNSFNGLINIGCTPINI*  Normal protein sequence |
| 7 | Hetan1.AOL_e_gw1.9.435.1 | \|  \| 181100 \| \| --- \| --- \| | scaffold_11:944602-945057 | >jgi\|Hetan2\|181100\|Hetan1.AOL_e_gw1.9.435.1  MFHRITLSILYLLFISLFASATPVTRWKGQCDAGPVQCCNSVQKSSNPSVAKILSGIAVPLQGLSVPIGLTCSPLNLLALGGNSCASQPVCCNNNFNGLVAIGCTPIDLGAL* Normal protein sequence |
| 8 | Hetan1.AOL_estExt_fgenesh3_kg.C_90132 | \|  \| 181098 \| \| --- \| --- \| | scaffold_11:994969-995774 | >jgi\|Hetan2\|181098\|Hetan1.AOL_estExt_fgenesh3_kg.C_90132  MFFRISTVFVVALATFAAASPAPWGAPPPTTTTHPPVTTTVTVTAPATTTTIPASQCNTGDAQCCNSVQSATAPAVTSLLGLLGIVLEDINVLVGLSCDPISVIGVGGGANCVQQPVCCENNNFNGLINIGCTPINIFL* Normal protein sequence |
| 9 | Hetan1.estExt_Genewise1.C_30567 | \|  \|  \| \| --- \| --- \| \|  \| 33224 \| | scaffold_06:277929-278625 | >jgi\|Hetan2\|33224\|Hetan1.estExt_Genewise1.C_30567  MFARISTLFVFFFLGLALMVSSTPTPSSESLIARDGQCNTGTLQCCNSVQSSSDPVTSLLLGLLGVVLGGIDIPIGIQCTPITVIGVGSGANCVQQPVCCTGNTFNGLVTVGCSPINLGL*  Normal protein sequence |
| 10 | Hetan1.e_gw1.3.836.1 | 46054 | scaffold_06:274681-275203 | >jgi\|Hetan2\|46054\|Hetan1.e_gw1.3.836.1  MFARISSISAVFFLGFALMVSATPTPSPEMLAARGGQCNTGPLQCCNSVQQADAPGAAQLLKTLGVVVQGTTTMVGINCSPIPILGAATGTKCTQQPVCCENNNYNGLVNIGCSPINGDL*  Normal protein sequence |
| 11 | Hetan1.Genemark.6987_g | 105914 | scaffold_11:421219-421683 | >jgi\|Hetan2\|105914\|Hetan1.Genemark.6987_g  MFSILALIPVIALITLASASPTKRTEPASQCDTAPVQCCDSVQSAGSPAAANELGLLGIVVQDLNIPVGLTCTPISVIGVGSGASCDASPVCCEDNSYNGVVAIGCVPVDLSL* Normal hydrophobin sequence |
| 12 | Hetan1.Genemark.6987_g | 105914 | scaffold_11:421219-421683 | >jgi\|Hetan2\|105914\|Hetan1.Genemark.6987_g  MFSILALIPVIALITLASASPTKRTEPASQCDTAPVQCCDSVQSAGSPAAANELGLLGIVVQDLNIPVGLTCTPISVIGVGSGASCDASPVCCEDNSYNGVVAIGCVPVDLSL  Normal protein sequence |
| 13 | Hetan1.ake_EuGene9000342 | \|  \| 181241 \| \| --- \| --- \| | scaffold_11:982306-983749 | >jgi\|Hetan2\|181241\|Hetan1.ake_EuGene9000342  MLTRISALSAVSILAVATMAAAGPVAADYGKPASYAPAADYGKPSSDYKPDYVPKDDYAPKEYGKDDYAPKEYGKDEYASQDDYSEDAYSKDVYPKDDYSKDDYSEDAYSKDAYPKDDYSKDAYPGDDYAPKEYGEYDYAPKKYGKDDYSKDEYAPKDYGKDDYSKDAYPNDDYSEDDYGKNPKEVYSKEAYGKESYEVFYKRDGGEGYGRGRGYGRKSFDKYGHHGLDDFDGDRFDGNDEYRGRDRYDDDEYRGRDRYDDDDDYRRGGDHYENSNYENNYESKCNAGPVQCCNTVEDVNSENAAQALGLLGLIIQGANIPIGLNCDPINLAAGFGSGSNCVSQPVCCDRVEGVRRRCDQLQSYQRRSLSTYQVIRGEERKERRGAEELLYPHTIFIVCLYSTPLYW*  Relatively long protein, parts of the protein are regions of unspliced introns, there are series of repeats sequences which may propably have inactivated the protein |
| 14 | Hetan1.AOL_EuGene18000072 | 181114 | scaffold_03:3245707-3246474 | >jgi\|Hetan2\|181114\|Hetan1.AOL_EuGene18000072  MVIKLSSFFLIALAASVVAAPRGEGLIPTIIPTVLPTDLPTSLPTGIPTDLPISLPSGIPTDLPTSLPSGIPTELPTSLPTGIPTDLPISLPSGIPTDLPTSLPVGLPTIIPTSIIPTITLSIPALPTGTLLPGSQCDTGPVQCCQSSGTAGDPGIASVLSLIGVVVEDLDVVVGVTCAPIDVVGLGSGATCDADPLCCEDNNFNSVVAIGCVPVNLAL*  Several repeat units |
| 15 | Hetan1.estExt_Genewise1.C_30567 | \|  \| 33224 \| \| --- \| --- \| | scaffold_11:359300-360496 | >jgi\|Hetan2\|33224\|Hetan1.estExt_Genewise1.C_30567  MFARISTLFVFFFLGLALMVSSTPTPSSESLIARDGQCNTGTLQCCNSVQSSSDPVTSLLLGLLGVVLGGIDIPIGIQCTPITVIGVGSGANCVQQPVCCTGNTFNGLVTVGCSPINLGL*  Normal protein sequence |
| 16 | Hetan1.e_gw1.3.836.1 | \|  \| 46054 \| \| --- \| --- \| | scaffold_06:274681-275203 | >jgi\|Hetan2\|46054\|Hetan1.e_gw1.3.836.1  MFARISSISAVFFLGFALMVSATPTPSPEMLAARGGQCNTGPLQCCNSVQQADAPGAAQLLKTLGVVVQGTTTMVGINCSPIPILGAATGTKCTQQPVCCENNNYNGLVNIGCSPINGDL  Normal protein sequence |
| 17 | Hetan1.Genemark.6987_g | 105914 | scaffold_11:421219-421683 | >jgi\|Hetan2\|105914\|Hetan1.Genemark.6987_g  MFSILALIPVIALITLASASPTKRTEPASQCDTAPVQCCDSVQSAGSPAAANELGLLGIVVQDLNIPVGLTCTPISVIGVGSGASCDASPVCCEDNSYNGVVAIGCVPVDLSL  Normal protein sequence |
| 18 | Hetan1.ake_EuGene9000342 | 181241 | scaffold_11:982306-983749 | >jgi\|Hetan2\|181241\|Hetan1.ake_EuGene9000342  MLTRISALSAVSILAVATMAAAGPVAADYGKPASYAPAADYGKPSSDYKPDYVPKDDYAPKEYGKDDYAPKEYGKDEYASQDDYSEDAYSKDVYPKDDYSKDDYSEDAYSKDAYPKDDYSKDAYPGDDYAPKEYGEYDYAPKKYGKDDYSKDEYAPKDYGKDDYSKDAYPNDDYSEDDYGKNPKEVYSKEAYGKESYEVFYKRDGGEGYGRGRGYGRKSFDKYGHHGLDDFDGDRFDGNDEYRGRDRYDDDEYRGRDRYDDDDDYRRGGDHYENSNYENNYESKCNAGPVQCCNTVEDVNSENAAQALGLLGLIIQGANIPIGLNCDPINLAAGFGSGSNCVSQPVCCDRVEGVRRRCDQLQSYQRRSLSTYQVIRGEERKERRGAEELLYPHTIFIVCLYSTPLYW*  A reasonable lenght of the gene was absent in other proteins, a closer look at the gene showed that this portion is lined with repeat units, the gene may not be functional. Part of the C-terminus contains unspliced intron |
| 19 | Hetan1.AOL_EuGene18000072 | 181114 | scaffold_03:3245707-3246474 | >jgi\|Hetan2\|181114\|Hetan1.AOL_EuGene18000072  MVIKLSSFFLIALAASVVAAPRGEGLIPTIIPTVLPTDLPTSLPTGIPTDLPISLPSGIPTDLPTSLPSGIPTELPTSLPTGIPTDLPISLPSGIPTDLPTSLPVGLPTIIPTSIIPTITLSIPALPTGTLLPGSQCDTGPVQCCQSSGTAGDPGIASVLSLIGVVVEDLDVVVGVTCAPIDVVGLGSGATCDADPLCCEDNNFNSVVAIGCVPVNLAL  A relatively long protein with most of the N-terminal region being inactivated by repeats |
| 20 | Hetan1.estExt_Genewise1Plus.C_90176 | \|  \| 65822 \| \| --- \| --- \| | scaffold_11:359300-360496 | >jgi\|Hetan2\|65822\|Hetan1.estExt_Genewise1Plus.C_90176  MFSRISAVSAVSFLTLAVVVSAGPWGSMKPEYKPVPAPVPTPEYTAAPAYTTTPYKDDYKEPEYPKTTAYAPAYPKDDYPSPAYPTDDYSSQEYPKDDYKYPEPVKDEYEPKKEEYKSDYLDDYRRRDYAPEPPKGYPVNDKPYADPKKEYDPKKEYDPKKPYDPKKEYDPKKPYDPKKEYDPKKEYDPKDKDYKSVKDVKDEYAHDIYNEEEHKMQCNSGPVQCCNTVEEARSEGAAKALGLLGIVLNDLNTPIGLNCDSLNAAGFGSGSNCVSQTVCCDRTEFGGLGINCSPINIVL*  A part of the protein is fused with an unknown sequence of which part of it contains several repeat units. |
| 21 | Hetan1.Genemark.5594_g | 104521 | scaffold_08:1919324-1919765 | >jgi\|Hetan2\|104521\|Hetan1.Genemark.5594_g  MSKLLRIVGLLVSACALARASSAATAQGSFSCDVGEAQCCANVYSPNDTRVGAVLKAIGLDASGAAVGVA  CDAMVPGGPSCEYTAACCTNNGFSGMVALGCARV*  Normal protein sequence |
| 22 | Hetan1.estExt_fgenesh3_kg.C_90130 | 148119 | scaffold_11:984272-985276 | >jgi\|Hetan2\|148119\|Hetan1.estExt_fgenesh3_kg.C_90130  MFARVSTLFAMFFLGLALMVSATPAALKPVARDTIPASQCNTGDLQCCNTVENADSPSAAALLGLLGVVVQGLDVLVGLTCTPITVIGVGSGANCVQQPVCCENNNFNGLINIGCTPVNLGL  Normal protein sequence |
| 23 | Hetan1.AOL_estExt_fgenesh3_kg.C_90057 | \|  \| 181099 \| \| --- \| --- \| | scaffold_11:423087-424783 | >jgi\|Hetan2\|181099\|Hetan1.AOL_estExt_fgenesh3_kg.C_90057  KGDCAIIIGT*HSTFATINGDLPLGYVCCAN*LSPEQRITQCPSPSS*TAM*RATHHLF*MFATSGNLDLTDHAALK*MRSRTFFELLAMKISACLDAPPNMNVIPEGHRKV*LFHYIL*NVYSV*C*PLVQMVRERCNRWNLTPIWQLSPAHRILDFVG*VEVCGVAFPLAEKLRRRMRRRGTRPSNSMNSSNLTRSS*RPPGVPCPDATKT*LHVWCH*GQRYFQR*KERPEVAFKR*VNSEIFWQIPCSIK*ACPPTVSSVISPTLDH*NVCQILSSCCDYLRRN*LCSRLGLAADPDHHRNSGPPADDDKSGQPMQHRPRAMLR*HHNGWRTGCCGCPVSPRRCRTGS*RGRRPLLLPDICCRSWEWCQLVSNLSRETGEIEAIDNNHL*RR*SRLLRLARWPHLHWLFPRQSRPLSSQKWPKEKVVTFMTKLCMESVKERRVVG*SSG*PALCCPLC*TCANAFSRLGYVVRSESGSKSGGHK*I  A  Appear to be a pseudogene |
| 24 |  |  |  |  |
| 25 |  |  |  |  |
