# Supplementary material for: Evolutionary analysis of hydrophobin gene family in two wood-degrading basidiomycetes, Phlebia brevispora and Heterobasidion annosum s.l
Source: BMC Evol Biol. 2013 Nov 4;13:240. doi: 10.1186/1471-2148-13-240 (PMC3879219; doi:10.1186/1471-2148-13-240)
Supplement: Additional file 7: Table S3 — Inventory of hydrophobin encoding genes in H. irregulare. [file 1471-2148-13-240-S7.docx]

Additional file 7: Table S3: Inventory of *Phlebia brevispora* hydrophobins

|  | Model name | Protein ID | Location | Remarks |
| --- | --- | --- | --- | --- |
| 1 | fgenesh1_kg.6_#_44_#_isotig12003 | 26991 | scaffold_6:175845-176392 | The existing protein sequence lacks N-terminal part. >jgi\|Phlbr1\|26991\|fgenesh1_kg.6_#_44_#_isotig12003  MPNNPPPVTTTITVTAPAPTVTTVSQCNTGSIQCCQQVESASSAAGSLLLGLLGVVLTDLDVLLGLDCSPISIIGVGSGSACSANPVCCENSSLGGLISIGCVPITL  I would suggest the following sequence with the N-terminal part corrected  >jgi\|Phlbr1\|26991\|fgenesh1_kg.6_#_44_#_isotig12003  MFARVYNTIIYLVFSLSLLAAAMPNNPPPVTTTITVTAPAPTVTTVSQCNTGSIQCCQQVESASSAAGSLLLGLLGVVLTDLDVLLGLDCSPISIIGVGSGSACSANPVCCENSSLGGLISIGCVPITL |
| 2 | fgenesh1_kg.6_#_39_#_isotig12003 | 26986 | scaffold_6:161334-161881 | The protein sequence lacks N-terminal part. >jgi\|Phlbr1\|26986\|fgenesh1_kg.6_#_39_#_isotig12003  MPNNPPPVTTTITVTAPAPTVTTVSQCNTGSIQCCQQVESASSAAGSLLLGLLGVVLTDLDVLLGLDCSPISIIGVGSGSACSANPVCCENSSLGGLISIGCVPITL.  I would suggest the following sequence with the N-terminal part corrected MFARVYNTIIYLVFSLSLLAAAMPNNPPPVTTTITVTAPAPTVTTVSQCNTGSIQCCQQVESASSAAGSLLLGLLGVVLTDLDVLLGLDCSPISIIGVGSGSACSANPVCCENSSLGGLISIGCVPITL |
| 3 | fgenesh1_kg.6_#_325_#_isotig09956 | 27272 | scaffold_6:1746245-1747097 | There is possibly an additional intron in the N-terminal part of the sequence  >jgi\|Phlbr1\|27272\|fgenesh1_kg.6_#_325_#_isotig09956  MFSRATAVVVSALAVLAAATPNPDNAKRWGVPTTSATVTTTITVTAPAPTVTTVSQCNTAPIQCCEQVVEGDSDAATAILGLLGIVLTDLDVLLGLNCSPITIIGVGTGNECSATPVCCENNSVGGLISIGCIPIIL  I would therefore suggest the following corrected sequence MFSRATAVVVSALAVLAAATPNPDNAKRWGVPTTQCNTAPIQCCEQVVEGDSDAATAILGLLGIVLTDLDVLLGLNCSPITIIGVGTGNECSATPVCCENNSVGGLISIGCIPIIL |
| 4 | fgenesh1_kg.11_#_148_#_isotig01503 | 28467 | scaffold_11:1133598-1134374 | Protein model looks fine. The protein has 9 cysteine residues  >jgi\|Phlbr1\|28467\|fgenesh1_kg.11_#_148_#_isotig01503  MFARIYASFFYVLCALSLLAVATPWGAPPTVTVTVTAPAPTVTTVSQCNTGSIQCCNQVLDSSSEEAGLL  LGLLGIVLEGITGLLGLGCSPISIIGIGSGSECSASPVCCENNSIGGLINIGCIPITL |
| 5 | fgenesh1_kg.1442_#_1_#_isotig12003 | 31442 | scaffold_1442:1475-2022 | The existing protein sequence lacks N-terminal part>jgi\|Phlbr1\|31442\|fgenesh1_kg.1442_#_1_#_isotig12003  MPNNPPPVTTTITVTAPAPTVTTVSQCNTGSIQCCQQVESASSAAGSLLLGLLGVVLTDLDVLLGLDCSPISIIGVGSGSACSANPVCCENSSLGGLISIGCVPITL*  Efforts to extend the existing model so as to incorporate the missing N-terminal part could not work. I would therefore suggest an alternative model in which the N-terminal part can be extended.>jgi\|Phlbr1\|35792\|gw1.1442.1.1  MFARVYNTIIYLVFSLSLLAAAMPNNPPPVTTTITVTAPAPTVTTVSQCNTGSIQCCQQVESASSAAGSLLLGLLGVVLTDLDVLLGLDCSPISIIGVGSGSACSANPVCCENSSLGGLISIGCVPITL |
| 6 | gw1.19.87.1 | 35681 | scaffold_19:734160-734550 | A reasonable part of the N-terminus was missing in the protein. A frame shift caused the end of the protein sequence before the stop codon  >jgi\|Phlbr1\|35681\|gw1.19.87.1  CNTGSIQCCNQVLDSSSEEANIILDLLGIVLDGVSELVGFDCSPITDGGVDGGASCSASPVCCSNNNIGGIISVGCSPITM  In the corrected model, the missing N-terminal part was corrected by extending the existing protein model.  MFASFNTSVLYVLGTLSILAVATPGGSPPTVTFTVTAPAPTATTISQCNTGSIQCCNQVLDSSSEEANIILDLLGIVLDGVSELVGFDCSPITDGGVDGGASCSASPVCCSNNNIGGIISVGCSPITM.  The sequence corresponds to the model #82663 |
| 7 | e_gw1.6.374.1 | 73171 | scaffold_6:1744553-1745166 | The existing model looks fine >jgi\|Phlbr1\|73171\|e_gw1.6.374.1  MFSRLSTLPVLALALLAVATPNPVEKRNNPCNTGSIQCCESVQSASSETVTNILGLLGVVLSDLDVLVGL  TCSPLSIIGVGSGSACTASPVCCENNSIGGLISIGCVPITL |
| 8 | e_gw1.9.114.1 | 75990 | scaffold_9:238918-240111 | The potein is abnormally long. A huge part of the protein is covered by intron. The existing protein model has 9 cysteine residues  >jgi\|Phlbr1\|75990\|e_gw1.9.114.1  MQIRAQLALLLVGLASIDTQARPCDESSSLTPTSTHHSHTRRPTTSYYSPHHGHHHYTSVVTETVTQPTTVTRTSTQTRTNTQSQTETVTIASATTYTDAYIPPTTSSYSHTYSHTYTSWTSSWTPSSSTSRFSTSTYPSSSPSATATGTPSGQCNTGPVQCCDSVQNASSPAAASILSGISVPIGAGVPVGLSCTPIGVLSGGSGANCASTPVCCEDNTYNGLINIGCVPIVIQL  I would suggest the following corrected sequence where the intron has been completely removed  1\|75990\|e_gw1.9.114.1  MQIRAQLALLLVGLASIDTQARPSGQCNTGPVQCCDSVQNASSPAAASILSGISVPIGAGVPVGLSCTPIGVLSGGSGANCASTPVCCEDNTYNGLINIGCVPIVIQL |
| 9 | e_gw1.9.200.1 | 75979 | scaffold_9:241146-241619 | The existing model looks fine, exons>jgi\|Phlbr1\|75979\|e_gw1.9.200.1  MFRSIVAIVVLAASAAVRATPATLFSRSQCDTGSVSCCNSVQPAGSDAANDALSGVLNIPVGLGVPIGLDCTPINVIGEGSGANCNASPVCCENNYNGGLIGVSCVPILLQG |
| 10 | e_gw1.117.4.1 | 87636 | scaffold_117:12547-12994 | The existing protein model has 9 cysteine residues.  >jgi\|Phlbr1\|87636\|e_gw1.117.4.1  MKFSRALAASVLALPCLAAATPLEARQDTSQCNTGSIQCCSQTESASSESASFLLGLLGIVLEDITALIGLDCSPISVIGVGSGSACTASPVCCSNTAVGGLIGIGCVPISI* |
| 11 | e_gw1.23.130.1 | 84623 | scaffold_23:541556-542100 | The existing model looks fine The existing protein model has 9 cysteine residues.  >jgi\|Phlbr1\|84623\|e_gw1.23.130.1  MKFSRALAASVLALPCLAAATPLEARQDPTSQCNTGSIQCCSQTESASSESASFLLGLLGIVLEDITALIGLDCSPISVIGVGSGSACTASPVCCSNTAVGGLIGIGCVPISI* |
| 12 | estExt_Genemark1.C_110310 | 162498 | scaffold_11:1117007-1117855 | The existing protein model has 9 cysteine residues  >jgi\|Phlbr1\|162498\|estExt_Genemark1.C_110310  MFARLYASFFYVLCTLSLLAVATPWGVTPTVTVTVTAPAPTVTTVSQCNTGSISCCDQVLDSSSEEANLLLGLLGIVLDGVTGLLGLDCSPISVIGVGSGSECSASPVCCENNSVGGLINIGCVPITL |
| 13 | gm1.7962_g | 150276 | scaffold_12:1121852-1122470 | The existing protein model has 9 cysteine residues  >jgi\|Phlbr1\|150276\|gm1.7962_g  MHFTSTVSCAAVLALLALPSFALSIPLKGRQDDSPCATGPLQCCQANYDVDSATGKTLLALHQQSFPEFD  TSSSGSGGGGVGVGCNPVLGSTCTASPNCCEANAILLSLGCIVITL* |
| 14 | gm1.8452_g | 150766 | scaffold_14:305090-305728 | The existing protein model has 9 cysteine residues.  >jgi\|Phlbr1\|150766\|gm1.8452_g  MFARLYASFFYALCTLSLLAVATPWGAPATTTITVTAPAPTVTTVSQCNTGSIQCCQQVFSPSSAEGGLI  LGLLDIVLEGLTGLLGLDCSSINVVGIGSGNDCTASPVCCTDNNVGGLISIGCVPITL* |
| 15 | gm1.11495_g | 153809 | scaffold_23:536450-537087 | The existing protein model has 9 cysteine residues.  >jgi\|Phlbr1\|153809\|gm1.11495_g  MFIRTASAFLCILIAFATLAAAIPKNRRWDTTSATPIPATTTVTVTAAAPTVTVTVDQCNTGSVQCCNVVTQAGSPLGNLLLGLLGIVVSDIDVLLGADCTPLSIGEVLGGATCTATPVCCEDNSVDTLISIGCIVIIL |
| 16 | gw1.757.1.1 | 32005 | scaffold_757:331-798 | Protein model has missing N-terminal part.  >jgi\|Phlbr1\|32005\|gw1.757.1.1  CNTGDIQCCDQVLESDSDAASLLLSLLGIAIEGVTGLIGFDCSPLTVIGVGSGSECSADPVCCENNSVGGLINIGCVPISL  I would therefore suggest the following sequence below in which the missing N-terminal part is replaced.>jgi\|Phlbr1\|32005\|gw1.757.1.1  MFARIYASFFCAMSTLSLLAVATPWAVTPTVTVTVTAPAPTATTISQCNTGDIQCCDQVLESDSDAASLLLSLLGIAIEGVTGLIGFDCSPLTVIGVGSGSECSADPVCCENNSVGGLINIGCVPISL |
| 17 | gw1.12.200.1 | 38299 | scaffold_12:69451-69803 | The N-terminal part was conspicuously missing  >jgi\|Phlbr1\|38299\|gw1.12.200.1  CDTGPIQCCESTESANSAAGAAILQSIGVVLQDPSVLLGLDCSPISVIGVGGSACTADPVCCENNSFGSLVSIGCVPVSI.  I would therefore suggest the following sequence below in which the missing N-terminal part is replaced.  >jgi\|Phlbr1\|77891\|e_gw1.12.200.1  MFTHLVAFTALVLPLLAVATPGNFVSRASCDTGPIQCCESTESANSAAGAAILQSIGVVLQDPSVLLGLDCSPISVIGVGSGSACTADPVCCENNSFGSLVSIGCVPVSI |
| 18 | gw1.12.275.1 | 40879 | scaffold_12:1128632-1129038 | The N-terminal part was conspicuously missing  >jgi\|Phlbr1\|40879\|gw1.12.275.1  ALPLLAVATPTPQSAGTCTTSPVSCCEATFQPTPEVANELSGLLGVVLDDITALVGLGCSPISVVGVGSGTACTQSPVCCTSNGGAISLGCVPVTV  I would therefore suggest a new model in which the N-terminal part is in replaced.  >jgi\|Phlbr1\|32149\|gw1.12.17.1  MQFSVAVLLALPLLAVATPTPQSAGTCTTSPVSCCEATFQPTPEVANELSGLLGVVLDDITALVGLGCSP  ISVVGVGSGTACTQSPVCCTSNGGAISLGCVPVTV |
| 19 | e_gw1.384.2.1 | 88435 | scaffold_384:1595-2035 | The N-terminal part was conspicuously missing  >jgi\|Phlbr1\|88435\|e_gw1.384.2.1  MPGGAPPTTKTVTVTAPAPTVTTVSQCNTGPIQCCDQVESASSAAGSLLLGLLGIVLSDLNVLLGLGCSPITVIGVGAGGACSASPVCCENNSVGGLISIGCIPIT  In the sequence below the missing N-terminal part is corrected.  >jgi\|Phlbr1\|88435\|e_gw1.384.2.1  MRSSRAYAVLAYVLFSLSFLAAAMPGGAPPTTKTVTVTAPAPTVTTVSQCNTGPIQCCDQVESASSAAGSLLLGLLGIVLSDLNVLLGLGCSPITVIGVGAGGACSASPVCCENNSVGGLISIGCIPITL |
| 20 | e_gw1.384.2.1 | 82773 | scaffold_19:25604-26137 | Existing model looks fine >jgi\|Phlbr1\|82773\|e_gw1.19.142.1  MFARLYASFFYVLYTLFLLAVATPWGAPATTTITVTAPAPTATTVSQCNTGSMQCCQEVFSPESAEGGLILGLLNMVVEGLTGLLGLGCSSINVIGIGSGNNCEVSTVCCMDNNVGGSLISIRCVPITL* |
| 21 | e_gw1.23.107.1 | 84591 | scaffold_23:524319-524762 | Alignment of the sequence with other paralogues of hydrophobins showed that the N-terminal part was missing in the existing model.  >jgi\|Phlbr1\|84591\|e_gw1.23.107.1  MPGGAPPPTTKTVTVTAPAPTVTTVSQCNTGPIQCCDQVESASSAAGSLLLDLLGIVLSDLNVLLGLGCSPITVIGIGAGGACSASPVCCENNSVGGLISIGCIPITL  I would suggest the following corrected sequence with N-terminal parts replaced  The suggested sequence matches with the model #120697, location : scaffold_23:524126-524929  MPASRAYAVLAYVLFSLSFLAAAMPGGAPPPTTKTVTVTAPAPTVTTVSQCNTGPIQCCDQVESASSAAGSLLLDLLGIVLSDLNVLLGLGCSPITVIGIGAGGACSASPVCCENNSVGGLISIGCIPITL |
| 22 | gm1.7573_g | 149887 | scaffold_11:1120439-1121131 | In the existing model, there is the presence of an intron at the C-terminal region of the protein. Furthermore, the existing protein model has 9 cysteine residues  >jgi\|Phlbr1\|149887\|gm1.7573_g  MFARIYASFFCAMCTLSLLAVATLWAVTLTVTVTVTAPAPTATTISQCNTGDIQCCNQVLESDSDAASLLLSLLGIAIKGVTGLIGFDCSPLTVIGVGSGSECFADSVCCENNSVMRRPHQHRLRPDLSLSAVRPYRDDTADSLALLSASSSVPVVEFMNDANSHPLLISSLYYNPKNLHTYA  I would suggest the following corrected sequence with the intron removed  MFARIYASFFCAMCTLSLLAVATLWAVTLTVTVTVTAPAPTATTISQCNTGDIQCCNQVLESDSDAASLLLSLLGIAIKGVTGLIGFDCSPLTVIGVGSGSECFADSVCCENNSGGLINIGCVPISL |
| 23 | gm1.7697_g | 150011 | scaffold_12:192117-192572 | Alignment of the sequence with other paralogues presented a sequence in which the N-terminal part appeared to be too short. The signal peptides lied at the region of the 1^st^ 18 amino acids  >jgi\|Phlbr1\|150011\|gm1.7697_g  MKAIALIVPLVLQTFAIATPAYRRDDGSNPCTTTPLQCCESTHTTDSALGKALLQLSGHEDYIGDGDIGIGCSPLGGNTCTEQPNCCNVTGTFITIGCVPINI. I would therefore say that the protein sequence is Ok. |
| 24 | gm1.7960_g | 150274 | scaffold_12:1119222-1119730 | The existing model is fine  >jgi\|Phlbr1\|150274\|gm1.7960_g  MQFLAVALLALPALASALAFDRRQTNSTCPVGPLQCCQDTVERDSATGKTLLQLSGKDDPGYGSLAVGCNPILGSTCVASPNCCTETGLIAIGCIPINL* |
| 25 | gm1.8450_g | 150764 | scaffold_12:1119264-1119684 | The existing model is fine. However, the existing protein model has 9 cysteine residues.  >jgi\|Phlbr1\|150764\|  MSPRFYASFWYALCTLSLALAVPQFGVPATTVTVTATAPTVTAVNECNTGFVECCTQVIASNSQTASLLLGLLSISLVGNPDLLGLGCSPIPIAGVASGTACTVAPVCCTTSLAGGFLYAGCTPIVL |
| 26 | gm1.12192_g | 154506 | scaffold_30:160940-161371 | The existing protein model has 6 cysteine residues.  >jgi\|Phlbr1\|154506\|gm1.12192_g  MFSRVLVLGALAFPLLAVATPAVVARDDIECCSSTIPASSAAAAPILATIGVVLQDLDVLLGLDCSPISVIGVGSGSECSSSPVTCSDGVIGGIGIGCVPISI |
